# Synthesis of High-Entropy Alloy Nanomaterials for Electrocatalytic Multi-Electron Transfer Reactions

**DOI:** 10.1021/acsnano.6c04801

**Published:** 2026-05-11

**Authors:** Xiang Meng, Yunhao Wang, Liang Guo, Fengkun Hao, Fu Liu, Juan Wang, Guozhi Wang, Mingzheng Shao, Chaohui Wang, Xiaoqing Huang, Zhanxi Fan

**Affiliations:** † Department of Chemistry, 53025 City University of Hong Kong, Kowloon, Hong Kong SAR 999077, China; ‡ Hong Kong Branch of National Precious Metals Material Engineering Research Center (NPMM), City University of Hong Kong, Kowloon, Hong Kong SAR 999077, China; § State Key Laboratory of Physical Chemistry of Solid Surfaces, College of Chemistry and Chemical Engineering, 12466 Xiamen University, Xiamen 361005, China; ∥ Hong Kong Institute for Clean Energy, City University of Hong Kong, Kowloon, Hong Kong SAR 999077, China; ⊥ City University of Hong Kong Shenzhen Research Institute, Shenzhen 518057, China

**Keywords:** high-entropy alloys, nanomaterials, electrocatalysis, fuel cells, electrosynthesis, carbon dioxide reduction reaction, nitrate reduction reaction, C−N coupling

## Abstract

Electrocatalytic multielectron transfer reactions are essential for clean energy conversion and the synthesis of value-added chemicals, yet their practical development is limited by the low selectivity, insufficient stability, and high cost of conventional catalysts. High-entropy alloys (HEAs), particularly in nanoscale forms, have emerged as a promising catalyst platform because their diverse local atomic environments, tunable electronic structures, and enhanced structural stability enable flexible regulation of complex reaction pathways. This review highlights the fundamental features, developmental evolution, and reaction-specific advantages of HEA nanomaterials to adapt to electrocatalytic multielectron transfer reactions. We emphasize how compositional complexity, morphology control, and local electronic modulation govern catalytic activity, selectivity, and durability. We further propose existing challenges and future opportunities. Overall, this review provides insights for the rational design of next-generation HEA electrocatalysts from a reaction-oriented and development-driven perspective.

## Graphical abstract

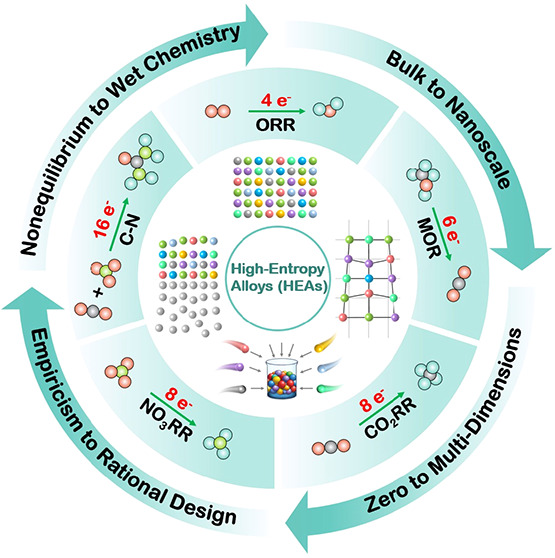

## 1 Introduction

With the continuous growth of global energy demand, the development of clean and renewable energy conversion technologies has become a core issue in contemporary scientific research.^1–3^ Electrocatalytic reactions, as highly efficient and environmentally friendly energy conversion processes, play a crucial role in fields such as fuel cells and electrosynthesis.^4–8^ Most of these reactions involve multielectron transfer processes, such as the oxygen reduction reaction (ORR, 4 electron transfer),^9^ methanol oxidation reaction (MOR, 6 electron transfer),^10^ carbon dioxide reduction reaction (CO_2_RR, 2–18 electron transfer, diverse products),^11^ and nitrate reduction reaction (NO_3_RR, 8 electron transfer).^12^ These multielectron transfer reaction pathways are complex, involving multiple intermediate adsorption, activation, and conversion steps, resulting in slow kinetics, high overpotentials, and poor selectivity.^13^ Traditional metal-based catalysts (Pt, Pd, Ir, etc.) exhibit excellent activity for specific reactions, but suffer from resource scarcity, high cost, insufficient stability, and catalyst poisoning, severely limiting their large-scale industrial applications.^14–18^

The complexity of electrocatalytic multielectron transfer reactions places higher demands on the active sites of catalysts. Single-component or traditional binary/ternary alloy catalysts can provide only a limited variety of active sites, making it difficult to simultaneously meet the adsorption energy requirements for different intermediates in multistep reactions.^19,20^ For example, for ORR, an ideal catalyst needs to simultaneously promote O–O bond breaking and O–H bond formation.^21^ For CO_2_RR, it is necessary to regulate the C–O and C–C bond coupling pathways to obtain high-value-added products (such as ethylene and ethanol).^22,23^ It can be seen that a single type of active site is insufficient to achieve multifunctional synergy. Therefore, there is an urgent need to introduce multicomponent catalysts with diverse surface electronic structures.^24,25^

High-entropy alloys (HEAs), as a novel type of multicomponent materials, offer a promising strategy to solving the aforementioned challenges.^26–30^ The concept of HEAs was first proposed in 2004.^28–31^ HEAs achieve a stable single-phase solid solution structure through high configuration entropy (Δ*S*_mix_ ≥ 1.5*R*).^32,33^ In addition, the formation of HEAs requires comprehensive consideration of the miscibility enthalpy, atomic radius differences, and synergistic effects among elements to ensure phase stability.^34^ HEAs possess four core effects: high-entropy effect, sluggish diffusion effect, lattice distortion effect, and cocktail effect.^35–37^ Among these, the interesting cocktail effect refers to the composite properties that exceed the simple sum of all elements by the synergy of multiple elements.^38,39^ These unique effects endow HEAs with excellent stability and corrosion resistance. In catalysis, HEAs also exhibit abundant surface active sites, continuously tunable electronic structures and excellent oxidation resistance.

The development of HEAs has evolved from bulk to nanoscale materials. Early research focused on bulk HEAs (such as the famous FeCrMnNiCo Cantor alloys), prepared via arc melting or mechanical alloying, emphasizing their mechanical properties.^28,40–42^ In recent years, driven by the electrocatalytic applications, HEA research has rapidly shifted to the nanoscale (such as nanoparticles (NPs), single-atom dispersions, and ultrathin nanosheets), achieving a transformation from unguided empirical synthesis to rational design.^43–45^ For example, wet chemical methods under mild conditions were developed to enable precise control over composition and morphology, as well as the synthesis of multidimensional structures with anisotropy.^46,47^ This evolution has significantly improved the specific surface area and active site exposure of HEAs, making them outstanding in electrocatalysis.

In all, HEAs provide an ideal platform for electrocatalytic multielectron transfer reactions due to their unique compositional flexibility, multisite synergistic effects, and high stability.^48^ This review systematically summarizes the latest advances in HEA nanomaterials for electrocatalytic multielectron transfer reactions. First, the basic principles and developments of HEAs are elucidated. Then, their applications in fuel cell-related reactions (e.g., ORR, MOR) and electrochemical synthesis (e.g., CO_2_RR, nitrogen reduction reaction/NO_3_RR (NRR/NO_3_RR), and C–N coupling) are discussed in detail. Finally, this review discusses challenges and prospects for future research, aiming to provide new ideas for the rational design of advanced HEA electrocatalysts.

Although HEAs have received widespread attention in the fields of catalysis and energy in recent years, most existing reviews focus on the basic characteristics of the materials systems themselves, or classify and discuss them around a specific reaction or a class of synthetic methods. There is a lack of a review that takes the commonalities of electrocatalytic multielectron transfer reactions as its theme, and systematically traces the development and methodological evolution of HEAs. First, current reviews generally lack a systematic summary based on the commonalities of electrocatalytic multielectron transfer reactions. Multielectron transfer reactions are inherently more challenging than simpler one/two-electron processes. Therefore, it is necessary to summarize the unique properties of HEAs in multielectron transfer reactions based on cross-reaction system comparisons. Second, in contrast to prior reviews, which often adopt a static and parallel structure, typically devoting separate sections to synthesis methods, morphology regulation, composition design, or individual applications without emphasizing interconnections, this review adopts a dynamic and developmental perspective. It highlights how synthesis methods, design strategies, and structural evolutions have progressed and mutually reinforced one another over time, thus revealing the universal advantages of HEAs for electrocatalysis.

## 2 Fundamentals of HEAs

### 2.1 Definition of HEAs

Traditional alloys typically involve one or two main elements as their basic framework, while HEAs are defined as alloys composed of five or more elements in (near-)­equiatomic or nonequiatomic ratios.^49^ In terms of elemental composition, it is generally believed that the proportion of each element in HEAs should be between 5% and 35%.^26,50^ However, with the expansion of high-entropy systems at the nanoscale, some HEAs break through this limitation. In addition, it is insufficient to prove the formation of single-phase solid solutions in multielement alloys based solely on the quantity and proportion of alloying elements. Thermodynamically, high mixing entropy, as the core driving force, significantly reduces the Gibbs free energy, inhibits the formation of brittle intermetallic compounds, and thus forms a stable solid solution phase. This characteristic of HEAs is typically defined by three key parameters: mixing entropy, mixing enthalpy, and atomic radius difference:^51^

#### 2.1.1 Mixing Entropy (Δ*S*_mix_)

Δ*S*_mix_ is the most crucial parameter for defining HEAs.^52^ For an alloy containing *n* elements, the formula for calculating configuration entropy is

$${\Delta S}_{\mathrm{mix}}=-R\sum_{i=1}^{n}C_{i}\ln C_{i}$$

where *C*_*i*_ is the atomic percentage of the *i*th element, and *R* is the molar gas constant. Based on the above formula as the classification standard, three ranges for materials were artificially defined: low-entropy (less than 1*R*), medium-entropy (1*R* – 1.5*R*), and high-entropy (greater than 1.5*R*). When the five elements are mixed in equiatomic ratios, Δ*S*_mix_ = 1.61*R*, which far exceeds the entropy of traditional materials. The high Δ*S*_mix_ reduces the Gibbs free energy (Δ*G* = Δ*H* – *T*Δ*S*), thereby stabilizing the solid solution phase.^53^

#### 2.1.2 Mixing Enthalpy (Δ*H*_mix_)

Δ*H*_mix_ reflects the strength of the interaction between different metal atoms. The formula for calculating configuration enthalpy is

$${\Delta H}_{mix}=4\sum_{i=1,i\neq j}^{n}{\Delta H}_{\mathrm{ij}}^{mix}C_{i}C_{j}$$

where Δ*H*_ij_^mix^ is the mixing enthalpy of binary liquid *ij* alloys, and *C*_*i*_ or *C*_*j*_ is the atomic percentage of the *i*th or *j*th element. Takeuchi and Inoue systematically and statistically analyzed the Δ*H*_mix_ for various binary alloys.^54^ A negative Δ*H*_mix_ indicates that atoms tend to form ordered compounds, while a positive value potentially leads to phase separation. For HEAs, the ideal range of Δ*H*_mix_ is typically between −15 and 5 kJ mol^–1^. A moderate Δ*H*_mix_, combined with a high Δ*S*_mix_, is the key to the formation of random solid solutions.^55,56^

#### 2.1.3 Atomic Radius Difference (δ)

Unlike the uniform lattice structure of pure metals, HEAs often exhibit significant lattice distortion due to atomic radius difference.^57,58^ δ could be calculated by

$$\mathrm{r̅}=\sum_{i=1}^{n}C_{i}r_{i}$$

$$\delta =\sqrt{\sum_{i=1}^{n}{C_{i}\left(1-\frac{r_{i}}{\mathrm{r̅}}\right)}^{2}}$$

where *r̅* is the average atomic radius of HEAs. The δ value is typically required to be less than 6.5%.^59^ Excessive δ can lead to severe lattice distortion, potentially compromising the stability of the solid solution. In other words, although creating some lattice distortions and defects may improve electrocatalytic performance, an appropriate δ is crucial for maintaining the stability of HEA structures.^60^

It is necessary to explicitly clarify the distinctions between true HEAs, multicomponent alloys, and high-entropy-inspired materials (or high-entropy systems), as these terms are sometimes used interchangeably in the broader literature, which can lead to conceptual confusion. Multicomponent alloys (typically containing three or more elements) share some compositional similarities with HEAs.^61,62^ For example, they both involve the introduction of multiple metallic elements to optimize synergistic performance and, in some cases, the formation of solid solution phases. Both systems aim to explore a broad compositional space and can benefit from interelement interactions, thereby enhancing catalytic performance and structural stability. However, they differ in fundamental thermodynamic drivers and microstructural characteristics. Multicomponent alloys typically exhibit lower configurational entropy (Δ*S*_mix_ is usually much lower than 1.5*R*), and their phase formation is primarily controlled by enthalpy contributions rather than entropy stability. Furthermore, the elemental ratios in multicomponent alloys are often not (near-)­equiatomic, with one or two dominant elements, which limits the full realization of the “core effect” of high-entropy systems, especially the high-entropy effect. In the field of electrocatalytic multielectron transfer reactions, multicomponent alloys typically possess more homogeneous but less diverse local atomic environments, resulting in a relatively discrete adsorption energy distribution. Therefore, they are less efficient at achieving multisite synergistic interactions. Essentially, multicomponent alloys can be considered as a broader extension of the high-entropy concept, but they lack a dominant effect. It also lacks the unique advantages of HEAs in complex reaction pathways involving multiple proton-coupled electron transfers.

Furthermore, high-entropy-inspired materials, on the other hand, represent an even broader extension of the high-entropy philosophy beyond metallic alloys into nonmetallic or compound systems, including high-entropy oxides, sulfides, nitrides, and intermetallics. These materials typically involve multiple cations or anions in the oxide/sulfide lattices, where entropy stabilization competes with strong ionic/covalent bonding and charge compensation effects. Due to the presence of nonmetallic elements and alterations in defect chemistry, their phase stability, electronic structure, and catalytic mechanisms differ fundamentally from those of metallic HEAs. For example, high-entropy oxides typically form rock-salt or spinel structures, functioning through oxygen-mediated charge transfer, while HEAs achieve electrocatalysis through metallic bonding and localized electrons, with their d-orbital centers being more easily tunable. While high-entropy-inspired materials show promise in electrocatalytic reactions, their intrinsic attributes place them outside the strict “alloy” designation and beyond the discussion scope of this review.

### 2.2 Core Effects of HEAs

Alloys that meet the above conditions tend to form unique structures with the “high-entropy effect” and exhibit superior properties not found in traditional alloys. It is worth noting that the fundamental characteristic of HEAs is not merely the increase in compositional complexity, but rather the generation of a series of unexpected phenomena at multiple levels, thermodynamics, kinetics, and electronic structures. These phenomena are systematically summarized as the “four core effects” (Figure 1).^36^

**Figure 1:**
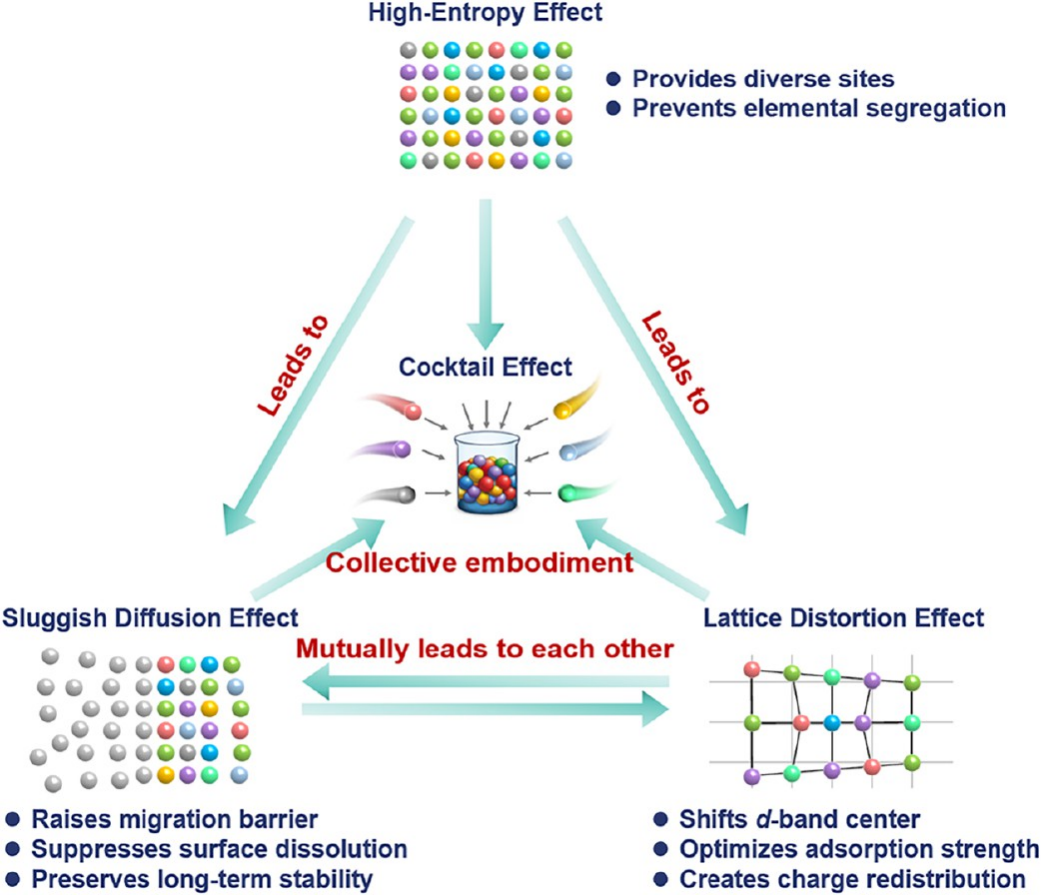
Schematic illustration of the core effects of HEAs.

#### 2.2.1 High-Entropy Effect

The high-entropy effect is the core effect of HEAs and the origin of their unique properties.^26,31,51,63^ It refers to the extremely high Δ*S*_mix_ of an alloy system when five or more elements are mixed in similar proportions. This high entropy can overcome elemental separation or high ordering caused by Δ*H*_mix_, thus directing the free energy toward the lowest state (disordered solid solution). The high-entropy effect could “anchor” multiple elements with different physicochemical properties within a simple crystal lattice, such as face-centered cubic (*fcc*), hexagonal close-packed (*hcp*), and body-centered cubic (*bcc*), suppressing the emergence of multiple complex crystal phases.^49^ Therefore, this high-entropy effect endows the catalyst with excellent structural stability, ensuring no elemental segregation in harsh electrocatalytic tests. Furthermore, the high-entropy effect allows for atomically homogeneous mixing of multiple elements. By fine-tuning the types and amounts of elements, accurate and continuous exploration of structure–activity relationships can be achieved. This differs from the traditional catalysts with limited and discontinuous active sites. In the future, high-throughput computing and artificial intelligence (AI) should be used to systematically screen and match active sites.

#### 2.2.2 Lattice Distortion Effect

The lattice distortion effect originates from the atomic radius differences in HEAs.^58^ When different atoms with various radii randomly occupy lattice sites, it leads to local lattice strain and distortion. The most critical consequence of lattice distortion is to change the electronic structure.^64^ Severe local lattice distortion can change the overlap integral between different atoms, resulting in a significant broadening of the d orbitals for transition metals, usually accompanied by the shift of the d-band center.^65,66^ It is well-known that the shift of the d-band center can modulate the adsorption behavior of intermediates on the catalyst surface, thereby optimizing reaction kinetics.^67–69^ Generally, the upward shift of the d-band center leads to enhanced metal-molecule interactions, while the downward shift leads to weakened binding forces.^70,71^ Furthermore, because different adjacent metal atoms have different electronegativity and work functions, the lattice distortion effect often induces uneven charge distribution. These changes in the electronic structure of HEAs caused by lattice distortion can significantly affect electrocatalytic performance. However, it should be noted that the lattice distortion degree should be moderate. Excessive lattice distortion can lead to lattice instability, inducing phase transitions, or even lattice collapse and amorphization. Therefore, achieving a balance between high activity (moderate distortion) and high stability (avoiding excessive distortion) is crucial. Future research should focus on using advanced in situ characterization techniques to accurately monitor the lattice distortion and electronic structure transitions of HEAs.

#### 2.2.3 Sluggish Diffusion Effect

The sluggish diffusion effect refers to the slower atomic diffusion efficiency in HEAs compared to traditional alloys.^49,51,72^ This originates from the nonuniform potential energy environment created by the random occupancy of multiple atoms and lattice distortion effect. When atoms tend to jump into vacancies, they must traverse migration paths with drastically fluctuating chemical bonding and strain field, significantly increasing the migration energy barrier. It can be seen that the sluggish diffusion effect is not significant in traditional alloys (mono/bimetallic) because the local energy environments of adjacent atoms are similar. While the sluggish diffusion effect does not directly regulate the electronic structure like the lattice distortion effect, it is a significant factor in improving creep resistance and suppressing elemental segregation. Therefore, this effect can improve electrocatalytic stability, greatly reduce the dissolution and diffusion of surface atoms. This allows HEA catalysts to maintain stable morphology, composition, specific surface area, and crystal phase over the long-term electrolysis.^73,74^

#### 2.2.4 Cocktail Effect

The cocktail effect refers to some unexpected and interesting properties arising from the synergistic effects of multiple components in HEAs.^50,51,75^ The term “cocktail” emphasizes its modulated nature. While sometimes referring to the unexpected physicochemical properties (such as tunable electronic structures and enhanced mechanical strength) after mixing multiple components, the cocktail effect is more commonly used to describe improved electrocatalytic performance in electrocatalysis field. Most importantly, its final manifestation is not a simple superposition of the properties of each component, but rather a superior comprehensive performance that surpasses any single component under complex interactions. This can manifest as increased catalytic activity, high selectivity for adsorption/reaction/desorption of reaction intermediates, and optimized reaction pathways. In other words, if the high-entropy effect, lattice distortion effect, and sluggish diffusion effect mainly reflect the intrinsic properties of HEAs, then the cocktail effect is the final manifestation of these three effects in terms of electrocatalytic performance. However, current research on the cocktail effect still faces some challenges. The mechanism of synergistic effects is unclear, and the exploration of structure–activity relationships is not in-depth enough. In the future, using machine learning (ML) and high-throughput computing to build predictive models can greatly reduce trial-and-error costs.

In all, these four core effects are not isolated but synergistic. The high-entropy effect is the core and prerequisite, thermodynamically ensuring the formation of a homogeneous solid solution. Lattice distortion is a consequence. The anchoring of multiple atoms within one lattice inevitably leads to distortion and defects. The sluggish diffusion effect and the lattice distortion effect are mutually causal. A suitable balance ensures the structural stability of HEA materials. The cocktail effect is the ultimate manifestation of performance through the unique structure constructed by the other three effects. In the field of electrocatalysis, the purpose of studying the four core effects of HEAs is to synergistically enhance electrocatalytic performance. Therefore, studying only one effect may be insufficient. And, due to their indivisibility, it is almost impossible to effectively isolate any one effect and study its contribution.

More importantly, in the field of electrocatalysis, the synergistic core effect of HEAs creates a catalytic environment different from that of traditional single-metal or simple alloy catalysts. The random distribution of multiple elements generates a large number of inequivalent local atomic configurations, resulting in a quasi-continuous distribution of adsorption energies. This characteristic is particularly important for complex multielectron transfer reactions, as these reactions require the sequential stabilization and transformation of multiple intermediates. In traditional catalysts, the adsorption energies of key intermediates are typically limited by linear scaling relationships, which fundamentally restricts activity and selectivity. In contrast, HEAs offer a wider adsorption mode, allowing different intermediates to preferentially interact with different sites, thus relaxing scaling constraints. Furthermore, the coexistence of chemically different but spatially adjacent sites enables multisite synergistic effects. In many multielectron transfer reactions, the fundamental steps do not necessarily require the same type of active site. Conversely, site A may be more favorable for the adsorption and activation of the initial reactant, and site B may promote proton/electron transfer, while another site C may promote product desorption or inhibit side reactions. Because HEAs themselves contain a large number of chemically distinct but spatially adjacent sites, they are particularly well-suited to support this multielectron transfer catalysis.

## 3 Developments of HEAs

### 3.1 From Bulk to Nanoscale

Early research on HEAs primarily focused on preparing bulk materials via vacuum casting and powder metallurgy, aiming to explore their potential for high strength, high toughness, and corrosion resistance.^76–78^ For example, the classic Cantor alloys (FeCrMnNiCo) exhibit excellent low-temperature toughness and corrosion resistance,^28,79^ while the refractory VNbMoTaW bulk alloys maintain a yield strength of over 400 MPa at 1600 °C, far superior to traditional Ni-based high-temperature alloys.^26,80,81^ However, the enormous volume, extremely low specific surface area, and unusable internal atoms of these bulk HEAs present significant bottlenecks for their electrocatalytic applications. On the one hand, a large number of active sites are wasted. Electrocatalytic reactions occur at the electrode/electrolyte interface.^82^ However, the reaction interface of bulk materials is extremely limited, and the vast majority of atoms cannot participate in the reaction. On the other hand, the mass transfer resistance during electrocatalysis is substantial. The diffusion paths of reactants and products through the surface of bulk materials are longer and more resistant, severely limiting reaction kinetics. Therefore, the nanoscale development of HEAs is a promising approach to overcome the above shortcomings of bulk materials. The evolution from bulk to nanoscale can result in an exponential increase in specific surface area and the number of surface active sites.^83^

For HEAs at the nanoscale, the proportion of surface atoms increases dramatically, leading to surface/interface dominance in material properties. This is crucial for improving electrocatalytic performance.^84–87^ In a typical study, He et al. reported a series of PtRuPdCoNi HEA nanomaterials with adjustable sizes (1.7, 2.3, 3.0, and 3.9 nm) (Figure 2a–d), and the ultrasmall HEAs with a size of 1.7 nm exhibit excellent performance and good stability in both hydrogen evolution reaction (HER) and ORR (Figure 2e).^88^ The kinetics of HEA NPs are closely related to their size. Furthermore, theoretical calculations show that differences in electronegativity among metals help optimize the electronic structure of HEAs (Figure 2f). The Bader charge describes the electron transfer from Co/Ni atoms to Pt/Ru/Pd atoms (Figure 2g). The overlapping d-band regions between different metals enhance the intermetallic interactions (Figure 2h). In another work, Guo et al. reported a class of IrRuRhMoW HEA NPs by choosing five metal carbonyl compounds (M­(CO)_*x*_, M = Ir, Ru, Rh, Mo, and W) as precursors via rapid codecomposition at 290 °C under argon (Ar) atmosphere (Figure 2i).^89^ The average size of HEA NPs is below 2 nm (Figure 2j). The IrRuRhMoW HEA NPs show a promising hydrogen oxidation reaction (HOR) kinetic current density of 8.09 mA μg_PGM_^–1^ at 50 mV (vs reversible hydrogen electrode (RHE)), which is 8.89 and 22.47 times that of IrRuRh NPs and commercial Pt/C, respectively.

**Figure 2:**
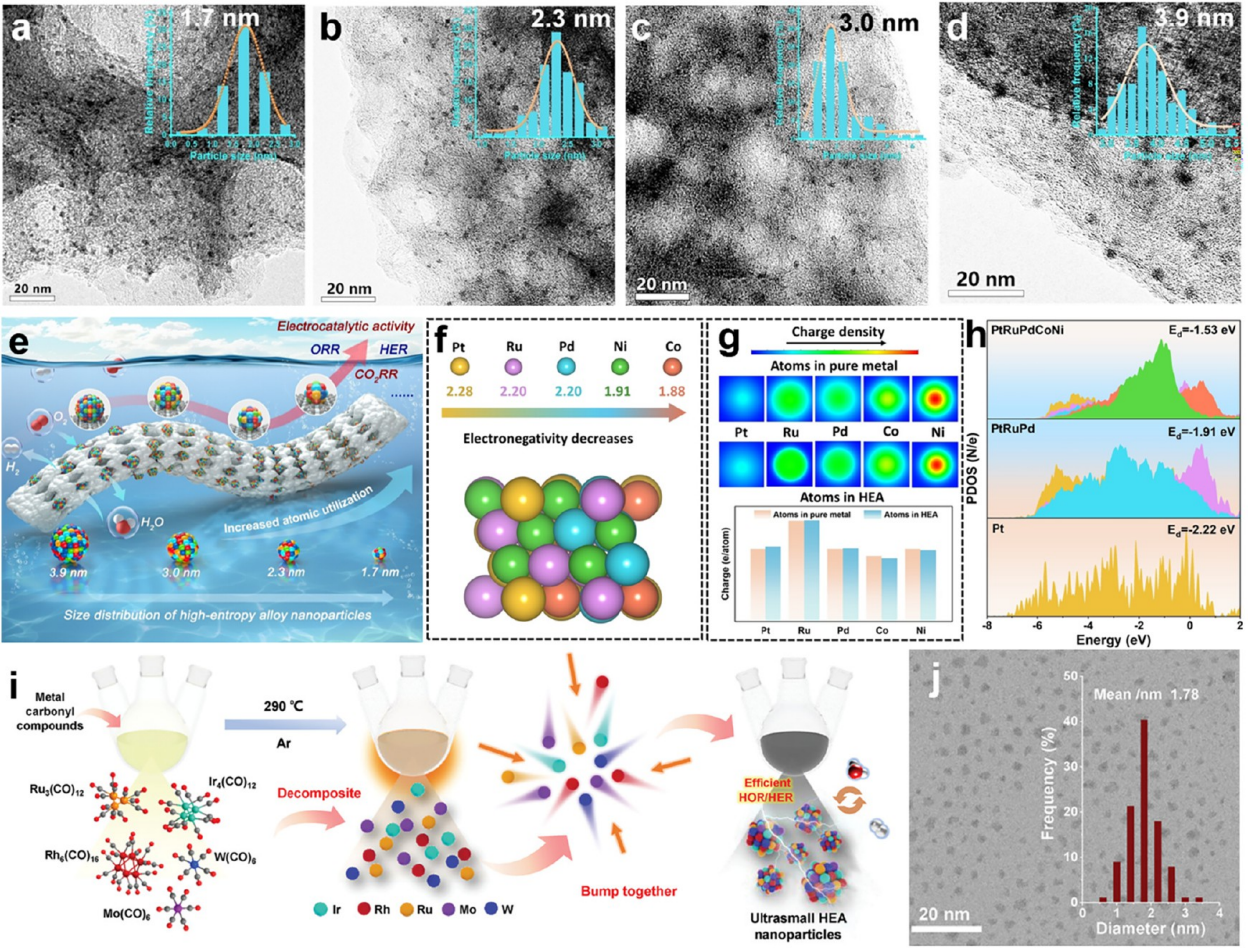
Nanoscale HEAs for efficient electrocatalytic applications. (a–d) Transmission electron microscopy (TEM) images of ultrasmall PtRuPdCoNi HEAs with four different sizes (Inset: particle size distributions). (e) Schematic illustration of size-tunable ultrasmall PtRuPdCoNi HEAs with a size-dependent electrocatalytic activity. (f) The electronegativity property for Pt, Ru, Pd, Ni and Co elements. (g) Two-dimensional charge density distributions of surface for pure metals and HEAs. (h) The d-band center of Pt sites for Pt(111), PtRuPd and PtRuPdCoNi HEAs. Reproduced with permission.^88^ Copyright 2025, Wiley-VCH. (i) Schematic illustration of the synthesis route of IrRuRhMoW HEA NPs. (j) TEM image of IrRuRhMoW HEA NPs (Inset: particle size distributions). Reproduced with permission.^89^ Copyright 2024, Wiley-VCH.

Furthermore, when the particle size of HEAs is reduced to the nanoscale, the quantum confinement effect (one of the major characteristics of nanomaterials) becomes prominent.^90^ The continuous energy bands can be discretized into quasi-atomic energy levels. Moreover, the coexistence of multiple elements makes this discretization process more complex, with intense hybridization and reconstruction of the electronic orbitals of different elements within the confined space. This enhances intrinsically the cocktail effect, and precisely adjusts the electronic structure such as the work function, d-band center, and electron distribution. In summary, the evolution from bulk materials to highly surface-active nanomaterials marks a fundamental change in structural design and catalytic function. By significantly increasing the proportion of accessible surface atoms, reducing transport resistance, and achieving size-dependent electronic structure tuning, nanoscale HEAs can overcome the inherent limitations of bulk HEAs and demonstrate greater potential in electrocatalytic applications. Currently, research on nanoscale HEA materials primarily focuses on developing synthesis methods.

### 3.2 From Empirical Development to Rational Design

Initially, the exploration of HEAs for electrocatalysis mainly involved the simple mixing of multiple elements in near-equiatomic ratios to achieve unexpected performance improvement. However, this research idea lacks rational design and often relies on trial and error. This undesigned or poorly designed approach has significant limitations. It is difficult to accurately predict active sites, guide reaction pathways, and control electronic structure as needed. Various HEA materials have been synthesized via simple methods and have exhibited promising electrocatalytic activity.^91^ However, the performance improvement is mainly attributed to the increase in the types and numbers of active sites resulting from the random mixing of multiple metals, rather than the rational control for specific reactions. Therefore, answering the questions of “why choose these elements” and “how to predict performance” is crucial.

On-demand element selection and combination are key to performance enhancement.^92^ The inherent properties of metal elements do not change easily. Therefore, when designing HEAs, the physicochemical properties of the elements need to be considered.^93,94^ For example, Hao et al. reported the importance of electronegativity differences among elements in the design of FeCoNiMnRu HEAs.^95^ Selecting metals with different electronegativity can effectively modulate the water dissociation barrier at every metal site (Figure 3a). For the materials synthesis, at a high temperature of 1000 °C, the ample energy supply causes metal atoms to diffuse rapidly, resulting in the uniform formation of a single-phase HEA (Figure 3b,c). Due to the charge redistribution, the most active Co and Ru sites can simultaneously stabilize *OH and *H intermediates (Figure 3d), significantly improving the HER performance in alkaline solution. For CO_2_RR, Cu species could effectively facilitate the C–C coupling to obtain C_2+_ products, and are regarded as a promising active site.^96,97^ By preserving the main active element (Cu) to the maximum extent, introducing some selected high-entropy doping elements (Ag/Au for high CO selectivity, and Sn/Bi for high HCOOH selectivity) can precisely control adsorption of specific intermediates.^98^ The “design-on-demand” strategy ensures that each element in the HEAs undertakes a specific function for guiding the reaction pathway, modulating the electronic structure, or stabilizing the structure.

**Figure 3:**
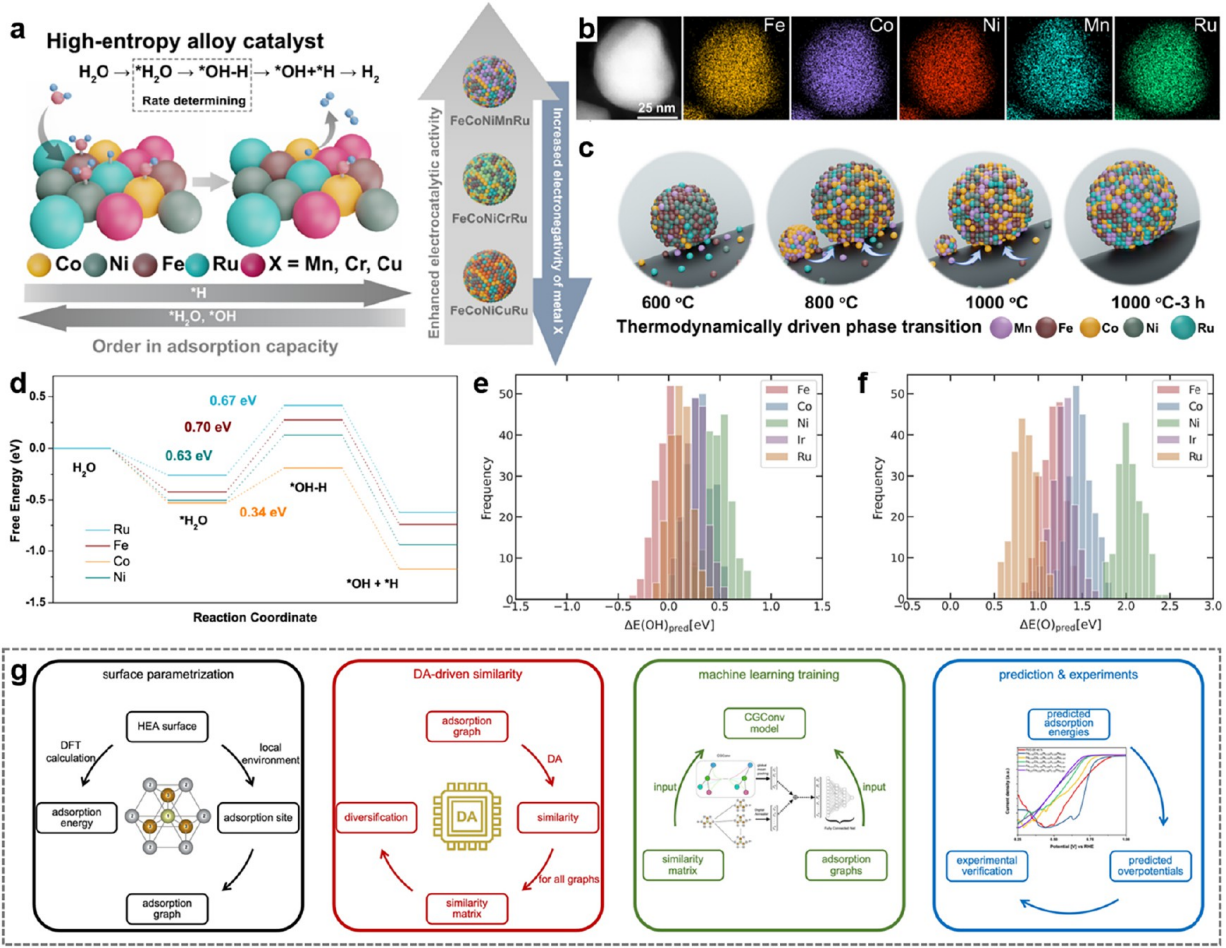
Rationally designed HEA nanomaterials for efficient electrocatalytic applications. (a) Schematic illustration of CoNiFeRuX (X = Mn, Cr, and Cu) HEAs with electronegativity-dependent preferences for the adsorption of the intermediates *OH and *H during H_2_O dissociation. (b) High-angle annular dark-field scanning TEM (HAADF-STEM) image and the corresponding STEM energy dispersive X-ray spectroscopy (STEM-EDS) elemental mappings of a FeCoNiMnRu HEA NP. (c) Schematic illustrations of the thermodynamically driven phase transition of FeCoNiMnRu HEA NPs on carbon nanofibers. (d) Free energy diagram for H_2_O dissociation at different catalytic sites (Ru, Fe, Co, and Ni) of FeCoNiMnRu HEAs. Reproduced with permission.^95^ Copyright 2022, Springer Nature. (e, f) ML-predicted (e) OH* and (f) O* adsorption energies for 1000 3 × 4 periodic unit cells of FeCoNiIrRu HEAs. (g) Computational flowchart for HEA electrocatalyst discovery. Reproduced with permission.^103^ Copyright 2024, Elsevier.

Furthermore, it must be emphasized that with the rapid development of computer science, computational tools such as density functional theory (DFT) calculations and ML have become key tools for guiding rational design.^99,100^ DFT calculations are often used to evaluate the adsorption energy of HEAs for various specific reaction intermediates, guiding and validating reaction pathways. For example, the Sabatier principle has been extensively studied in heterogeneous catalysis and is visualized by a volcano diagram.^101^ The optimal catalytic activity (i.e., adsorption energy) is located at the peak of the volcano diagram, and DFT calculations can be used to locate the HEA materials at this peak.^102^ ML, on the other hand, can build predictive models from descriptors to catalytic performance via high-throughput screening, which can then guide the synthesis of HEAs for specific reactions. Recently, Sargent et al. discovered a type of HEAs, Fe_0.125_Co_0.125_Ni_0.229_Ir_0.229_Ru_0.292_, as a promising ORR catalyst, utilizing ML to streamline the computational demands of DFT calculations.^103^ They hypothesized that introducing a quantitative measure of similarity among adsorption sites could improve accuracy when training a ML model on DFT data. Figure 3e,f show the distribution of ML-predicted OH* and O* adsorption energies. The workflow they proposed can reduce the required training data set size from 1600 to 800, resulting in a 2-times acceleration (Figure 3g).

It is worth noting that the best application of the above computational tools (i.e., DFT and ML) lies in the collaboration with experiments (including advanced in situ characterizations).^104^ Computation could predict the potential HEA materials, and guide experimental studies. Conversely, experimental results can validate and correct computational models, optimizing computational accuracy. In short, combining experimental and computational investigations to rationally design advanced HEAs is extremely important.

### 3.3 From Zero to Multidimensional Anisotropic Structures

Currently, the morphology of HEA nanomaterials is mainly limited to the zero-dimensional (0D) NPs.^16,85,105,106^ These 0D HEAs are usually prepared via various nonequilibrium synthesis methods, such as carbothermal shock and Joule heating.^107,108^ The nonequilibrium methods are characterized by ultrafast heating and cooling. This extreme condition can ensure rapid miscibility between multiple elements, forming a single-phase solid solution. However, after rapid nucleation, atoms do not have enough time for anisotropic diffusion. Therefore, HEA nanomaterials synthesized by these methods are typically near spherical. Moreover, under thermodynamic control, nanomaterials tend to form 0D NPs. 0D HEA NPs are widely used in electrocatalysis due to their high specific surface area that provides abundant active sites, thereby exhibiting good performance. However, they also face some challenges. NPs are prone to aggregation, and their high interfacial resistance limits electron transport. Meanwhile, NPs can also easily detach from the electrode in electrocatalysis due to the lack of a stable supporting structure.

Therefore, researchers try to design and synthesize anisotropic multidimensional nanostructures, including one-dimensional (1D) nanowires (NWs), two-dimensional (2D) nanosheets/nanoribbons, and three-dimensional (3D) structures.^86,109–112^ In a typical study, Guo et al. developed a mild general reduction-diffusion method at low-temperature for building a material library of atomic-thick Pt-based 1D HEA NWs (Figure 4a–c).^113^ With this method, 17 kinds of HEA NWs were successfully prepared. HEA NWs exhibit significant lattice distortion, which can modulate strain distribution and electronic structure, thus resulting in excellent electrocatalytic HOR and HER performance. In another work, Guo et al. also reported a class of PdPtNiCuZn 2D HEA nanosheets via the one-pot liquid-phase synthesis (Figure 4d).^114^ The average thickness of HEA nanosheets was determined to be approximately 1.69 nm using atomic force microscopy (AFM) (Figure 4e). More importantly, by being modified with Mo single atoms, the electronic structure and reaction pathway of Mo_1_–PdPtNiCuZn are adjusted. The Mo_1_–PdPtNiCuZn HEAs exhibit an excellent mass activity of 24.55 A mg_Pt_^–1^ toward MOR, 18.13 times that of commercial Pt/C catalysts. 3D nanomaterials (especially porous structures) are attracting greater attention due to their higher specific surface area exposure and superior gas/ion diffusion. Xia et al. innovatively constructed the 3D NiCoMoZnCu HEA nanoflower array (HEANFA) electrodes composed of 2D nanosheets with abundant active sites (Figure 4f).^115^ Atomic-resolution structural characterizations confirm the single-phase solid solution structure (Figure 4g). With exposed high-activity (111) facets and a superaerophobic surface (Figure 4h), HEANFAs demonstrate the top-level HER and hydrazine oxidation reaction performance. The self-supporting properties of multidimensional structures can effectively resist the aggregation of nanomaterials. Furthermore, the anisotropic multidimensional HEA materials can expose specific crystal facets with higher intrinsic activity. For example, Kang et al. prepared a kind of octahedra nanocrystals, PtFeNiCuW HEAs doped with Ru (Figure 4i).^116^ Such single-phase Ru-PtFeNiCuW HEAs are dominated by (111)-terminated facets (Figure 4j,k). In addition to enhancing catalytic activity, the corrosion resistance of Ru-PtFeNiCuW/CNTs catalysts is also significantly improved due to the exposure of specific (111) facets. HEA catalysts could work steadily for up to 1500 and 1200 h in acidic and alkaline electrolytes, respectively, at a current density of 50 mA cm^–2^ for full water splitting. In the electrolyte, the dissolution rate of nanomaterials with exposed (111) facets is an order of magnitude slower than that with exposed (100) facets.^117^

**Figure 4:**
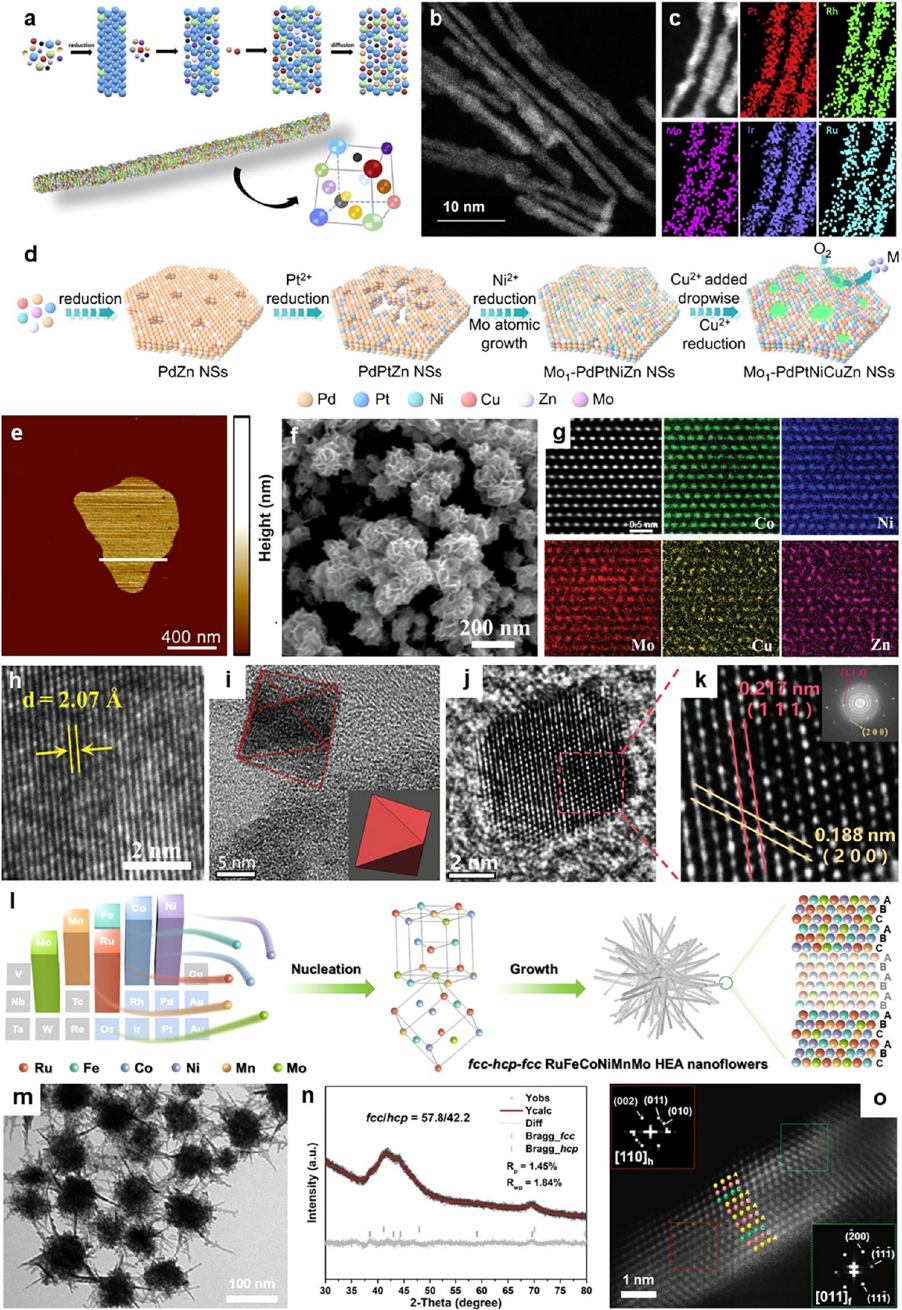
Multidimensional anisotropic HEA nanostructures. (a) Schematic illustration of the synthesis of HEA NWs with lattice distortion. (b) HAADF-STEM image and (c) STEM-EDS elemental mappings of PtRhMoRuIr HEA NWs. Reproduced with permission.^113^ Copyright 2023, Elsevier. (d) Schematic illustration of the synthesis route of Mo_1_–PdPtNiCuZn HEA nanosheets. (e) AFM image of a Mo_1_–PdPtNiCuZn HEA nanosheet. Reproduced with permission.^114^ Copyright 2024, Springer Nature. (f) Scanning electron microscopy (SEM) image, (g) atomic-resolution STEM-EDS elemental mappings of a magnified region, and (h) high-resolution TEM (HRTEM) image of HEANFA. Reproduced with permission.^115^ Copyright 2025, Wiley-VCH. (i) TEM and (j, k) HRTEM images of Ru-PtFeNiCuW/CNTs. The inset of (k) is the corresponding fast Fourier transform pattern. Reproduced with permission.^116^ Copyright 2024, Wiley-VCH. (l) Schematic illustration of the synthesis of heterophase *fcc*-*hcp*-*fcc* RuFeCoNiMnMo HEA nanoflowers. (m-o) TEM image (m), XRD pattern (n), and atomic resolution HAADF-STEM image (o) of heterophase *fcc*-*hcp*-*fcc* RuFeCoNiMnMo HEA nanoflowers. Insets of (o): the corresponding fast Fourier transform patterns of the areas marked by red and green dashed squares in (o). Reproduced with permission.^86^ Copyright 2026, Wiley-VCH.

As mentioned above, in the evolution of HEAs into multidimensional nanomaterials, the controllable synthesis of anisotropic morphologies has become a key strategy for exposing specific crystal facets, enriching edge/defect sites, and optimizing the local coordination environment, thereby significantly improving the activity and selectivity in electrocatalytic reactions. In particular, it is important to emphasize that crystal phase modulation is a crucial means of achieving anisotropic growth. By switching the crystal phase, such as *fcc*, *bcc*, and *hcp*, the atomic arrangement and surface electron distribution can be directly affected, thereby regulating the adsorption behavior for key intermediates.^118,119^ However, unlike traditional alloys, HEAs typically tend to form a conventional single-phase due to their thermodynamic stability dominated by high configuration entropy. Achieving controllable crystal phase modulation (e.g., *fcc* → *bcc* or *fcc* → *hcp*) remains significant challenges. Recently, Meng et al. developed the controllable synthesis of heterophase *fcc*-*hcp*-*fcc* RuFeMMnMo (M = CoNi, Co, and Ni) HEAs via the facile one-pot wet-chemical method (Figure 4l).^86^ A series of acetylacetonate and carbonyl metal salts was selected as precursors. Molybdenum hexacarbonyl (Mo­(CO)_6_), as a reducing agent, plays an important role in controlling the crystal phase. The synthesized *fcc*-*hcp*-*fcc* RuFeCoNiMnMo HEAs exhibit the 3D nanoflower structure assembled by ultrathin nanodendrites (Figure 4m). The X-ray diffraction (XRD) refinement analysis showed that the ratio of the *hcp* and *fcc* phase is 42.2/57.8 (Figure 4n). Importantly, as can be seen from HAADF-STEM image (Figure 4o), the middle region of the crystal exhibits a typical “AB” atomic stacking sequence along the close-packed [001]_h_ direction, while the two ends exhibit “ABC” atomic stacking sequence along the close-packed [111̅]_f_ direction, which proves the existence of *fcc*-*hcp*-*fcc* heterophase.

Currently, many anisotropic multidimensional materials have shown excellent electrocatalytic performance, but controllable synthesis remains a challenge. To synthesize specific structures, milder wet-chemical methods are increasingly preferred over nonequilibrium synthesis. However, this method may lead to elemental segregation in HEAs due to the differences in the reduction/diffusion rates of different elements. This may result in a core–shell structure instead of a simple solid solution.^120^ In addition, for binary or ternary alloys, certain surface modifiers and capping agents (generally small organic molecules) can directionally induce the growth of specific structures and crystal facets. However, high-entropy systems containing multiple metal precursors could disrupt the nucleation and growth processes. Nevertheless, anisotropic multidimensional HEA nanomaterials have great potential to improve electrocatalytic performance.

### 3.4 From Nonequilibrium to Wet-Chemical Synthesis

The synthesis strategies of HEAs have evolved from early thermodynamically nonequilibrium synthesis to the controllable wet-chemical synthesis.^121^ This evolution has not only facilitated the leap from bulk to nanoscale but also improved the morphological controllability of HEA materials, laying the foundation for their application in electrocatalysis. The nonequilibrium synthesis technique is the common method for synthesizing bulk HEAs, the most classic being vacuum arc melting. The classic Cantor alloy was prepared via this method.^28,122^ However, since bulk HEAs are not the focus of this review, they will not be described in detail here.

With the development of HEAs toward the nanoscale, a series of nonequilibrium synthesis methods have been rapidly expanded (Figure 5). In 2018, Hu et al. reported a groundbreaking carbothermal shock method.^107^ This method employs flash heating and cooling, in which the heating temperature is ∼2000 K, shock duration is ∼55 ms, and the heating/cooling rate is up to ∼10^5^ K/s (Figure 5a). Ultrafast heating drives the rapid “fission” and “fusion” of NPs, ultimately forming a homogeneous mixture of multiple elements. A subsequent fast cooling process could regulate the kinetics, promoting the formation of solid solutions. It is worth emphasizing that the carbothermal shock method has excellent versatility. Because its highest temperature (2000 to 3000 K) exceeds the decomposition temperatures of most metal salts, it can achieve the homogeneous mixing of most metal combinations. Initially, carbothermal shock relied on carbon nanofibers or carbon black as conductive/thermal carriers. Later, graphene (formed in situ after carbothermal shock),^123^ carbon nanotubes,^124^ and metal oxide^125,126^ carriers were developed, further improving particle dispersibility and interfacial electron transfer efficiency. Furthermore, the flash-thermal shock method was proposed using the thermal energy generated by the photothermal effect (energy exchange between photons and electrons in materials). Cha et al. reported that a single flash irradiation on CNFs could induce instantaneous high-temperature annealing (temperature rises to above 1800 °C within 20 ms, heating/cooling rate >10^4^ K/s), thereby successfully preparing HEAs with nine elements.^127^ A significant advantage of flash-thermal shock compared to carbothermal shock is that it can be operated in an air environment, rather than the Ar or vacuum atmosphere.

**Figure 5:**
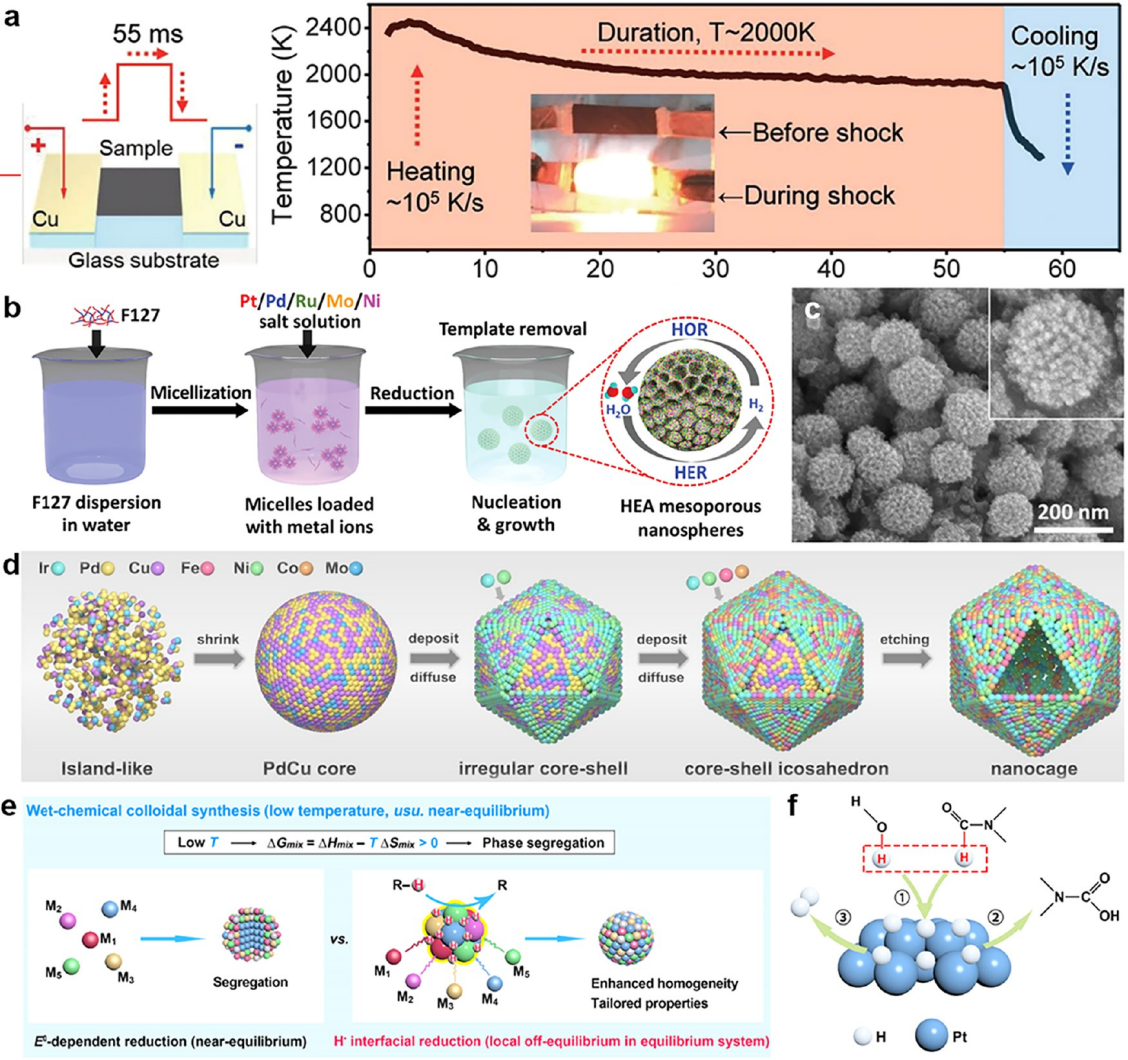
Multiple synthesis methods for HEA nanomaterials. (a) Schematic illustration of the carbothermal shock synthesis and the temporal evolution of temperature during the 55 ms thermal shock. Reproduced with permission.^107^ Copyright 2018, The American Association for the Advancement of Science. (b) Schematic illustration of mesoporous PtPdRuMoNi HEA nanospheres via one-pot wet-chemical synthesis. (c) SEM image of PtPdRuMoNi HEA nanospheres. Reproduced with permission.^133^ Copyright 2024, Wiley-VCH. (d) Schematic illustration of the synthesis mechanism of PdCu@IrPdCuFeCoNiMo and IrPdCuFeCoNiMo-NCs. Reproduced with permission.^134^ Copyright 2025, Wiley-VCH. (e) Schematic illustration of the wet-chemical colloidal synthesis. The H^•^ interfacial reduction approach creates a localized nonequilibrium environment for the reduction of multiple metal precursors. (f) Proposed H^•^ formation mechanism by the DMF dehydrogenation reaction. Reproduced with permission.^136^ Copyright 2025, American Chemical Society.

Besides carbothermal shock, Joule heating,^128^ physical vapor deposition,^129^ spray pyrolysis,^130^ and plasma discharge^131^ are also common nonequilibrium synthesis methods. These methods share the common characteristic of enabling the complete fusion of multiple elements in an extremely short time through ultrafast energy input and ultrafast cooling. The heating rate directly determines whether multiple elements can enter a “competitive but synchronous” reaction state. If the heating rate is low, different precursors will decompose and be reduced sequentially according to their respective thermodynamic and kinetic characteristics, ultimately forming phase-separated products. Conversely, ultrafast heating effectively levels out the originally significant reaction time differences between different elements in a short period. The ultrafast cooling process is the key kinetic process that determines whether the final structure can be preserved. The homogeneous mixed state formed at the high-temperature stage is a metastable state that is not thermodynamically completely stable. If cooling rate is fast enough, atoms do not have time for long-range diffusion and rearrangement, and this high-temperature mixed state can be “frozen” into a single-phase solid solution that can be retained at room temperature. Conversely, the system will be dominated by enthalpy again, resulting in the precipitation of intermetallic compounds. In this process, the role of precursor chemistry is equally important, and in many cases, it determines whether a nonequilibrium strategy can truly achieve effective homogeneous mixing of multiple elements. The coordination environment, decomposition pathway, volatility, reduction ease, and interaction with the support or solvent of different precursors all affect their reaction during instantaneous heating. However, their drawbacks are also obvious: difficulty in morphology control (mostly spherical NPs), low yield, and high energy consumption.

With the increasing demand for specific nanomaterial morphologies in electrocatalytic applications, wet-chemical strategies, typically used for the synthesis of noble metal nanocrystals, are being applied to the synthesis of HEAs.^46^ By selecting appropriate solvents, reducing agents, surface modifiers, capping agents, and reaction temperatures and times, HEAs can be synthesized under low-temperature conditions. For example, Prasad et al. explored various combinations of solvents, reducing agents, and capping agents in an attempt to synthesize pure-phase HEA.^132^ The results showed that not all combinations could reduce all metal elements to form HEA. Among the tested combinations, specific ratios of mixed solvents (benzyl alcohol and oleylamine) were favorable for the formation of single-phase PtPdCoNiMn HEA. Kitagawa et al. synthesized the HEA NPs by introducing a mixed aqueous solution of six platinum-group metals (PGMs, i.e., Ru, Rh, Pd, Os, Ir, and Pt) into the triethylene glycol solution (TEG) at a temperature of 230 °C.^106^ Poly­(*N*-vinyl-2-pyrrolidone) (PVP, K30, *M*_w_ ≈ 40,000) was added as the protecting agent, and TEG acted as the reducing agent and solvent. Most importantly, the advantage of wet-chemical methods is to prepare a series of special structures with anisotropic properties, such as porous, hollow, and nanoframework-like structures.^133^ Nandan et al. developed a one-pot wet-chemical strategy to produce mesoporous HEA nanospheres of PtPdRuMoNi, using a triblock copolymer as the soft template (Figure 5b,c).^133^ The mesoporous PtPdRuMoNi HEA structure could facilitate the exposure of multiple active sites, accelerate reaction kinetics, and contribute to improved catalytic HER and HOR performance. Long et al. reported a kind of IrPdCuFeNiCoMo HEA nanocages (IrPdCuFeNiCoMo-NCs).^134^ The hollow structure was realized by selective chemical etching of PdCu core (Figure 5d). The “generate first and then etch” strategy is the unique feature of wet-chemical methods. Interestingly, this IrPdCuFeNiCoMo-NC is icosahedra enclosed by (111) facets. This indicates that, in addition to morphology, HEAs synthesized via wet-chemical methods also could focus on aspects such as exposed crystal facets, defects,^135^ and sizes.^46^ However, this structural controllability sometimes comes at the cost of sacrificing some component homogeneity and phase stability. While wet-chemical methods offer precise control over composition and morphology, and the mild reaction conditions, their drawbacks are also significant. The large differences in redox potentials between various metals can easily lead to phase separation rather than a single-phase solid solution. This is the key trade-off between the wet-chemical and nonequilibrium methods: the former can achieve precise control over size, morphology, and interface structure under relatively mild conditions, while the latter is generally better at overcoming the differences in reduction behavior between different metal precursors, “freezing” multiple elements within the same lattice through ultrafast heating/cooling, thus making it easier to obtain homogeneous single-phase HEAs. For systems seeking atomically uniform distribution, nonequilibrium methods still have irreplaceable advantages. In short, the wet-chemical method is better at “shaping structures”, but it is often less effective than nonequilibrium strategies in ensuring that all elements are truly mixed at the atomic scale. Therefore, Zhang et al. presented that by utilizing the in situ dehydrogenation reaction of organic molecules (*N*,*N*-dimethylformamide, DMF) to generate highly active hydrogen (H^•^, i.e., hydrogen atoms or radicals), a localized off-equilibrium reducing environment is created in a near-equilibrium wet-chemical system within low-temperature of 170 °C (Figure 5e).^136^ The active H^•^ in the system is crucial for ensuring the simultaneous coreduction of multiple metals because the standard reduction potential of H is much lower than that of many metals (Figure 5f). The as-synthesized HEAs, represented by PtCuNiCoFe NPs, exhibit excellent MOR performance. The significance of this strategy lies in its attempt to simultaneously combine the structural controllability of wet chemistry with the coreduction advantages of nonequilibrium methods.

It is worth noting that the evolution from nonequilibrium to wet-chemical methods does not represent the replacement of outdated methods by more advanced ones. Rather, it simply means that the controllable nature of wet-chemical synthesis demonstrates flexibility in preparing anisotropic HEA nanomaterials with specific structures, which is beneficial for improving electrocatalytic activity.

## 4 Electrocatalytic Applications of HEAs

In the preceding sections, we systematically discussed the unique properties and development trends of HEA materials. These breakthroughs at the materials level, particularly the diversification of nanoscale morphologies, have laid a solid foundation for the application of HEA nanomaterials in electrocatalysis. This section will focus on reviewing the latest research progress on HEA nanomaterials for electrocatalytic multielectron transfer reactions (typically referring to electrocatalysis involving three or more electron transfers). Multielectron transfer reactions involve multistep reaction pathways, multiple reaction intermediates, and complex competing reactions. In contrast, two-electron transfer reactions (such as HER) have relatively simple reaction pathways, and the unique advantages of HEAs are not particularly prominent. This section presents several typical multielectron transfer reactions directly related to the hydrogen economy, carbon neutrality, and the green nitrogen cycle (Table 1). These reactions are all particularly promising for achieving clean energy conversion, and the sustainable synthesis of high-value-added chemicals and materials.

**Table 1:** Electrocatalytic Applications of the State-of-the-Art HEA Nanomaterials in Some Representative Multi-Electron Transfer Reactions

catalysts	structures	reactions	activity/selectivity	stability	refs
N-doped PtCoFeNiCu/C (N–Pt/HEA/C)	Core–shell	ORR	1.34 A mg_Pt_^–1^ at 0.9 V vs RHE	79.1% retention after 30,000 cycles	^137^
HEA-PtPdIrRuAg SNRs	Nanoribbon	ORR	4.28 A mg_Pt_^–1^ at 0.9 V vs RHE	56.5% retention after 30,000 cycles	^138^
PtPdFeCoNi HEA NRs	Nanoring	ORR	0.99 A mg_PGM_^–1^ at 0.95 V vs RHE	62.5% retention after 50,000 cycles	^66^
AlCuNiPtMn	Porous	ORR	3.5 A mg_Pt_^–1^ at 0.9 V vs RHE	30,000 cycles	^139^
AlNiCuPtPdAu np-HEA	Porous	ORR	2.24 A mg_Pt_^–1^ at 0.9 V vs RHE	100,000 cycles	^140^
PtFeCoNiMn/OMC	Nanoparticle	ORR	1.12 A mg_Pt_^–1^ at 0.9 V vs RHE	30,000 cycles	^91^
CoFeNiCuPd (OHEA-mNC)	Nanoparticle	ORR	2.037 mA μg_Pd_^–1^ at 0.9 V vs RHE	10 mV decay after 10,000 cycles	^141^
Mo_1_–PdPtNiCuZn	Nanosheet	MOR	24.55 A mg_Pt_^–1^	Strong stability after 10 h	^114^
PtNiCuMoCoIr/C	Nanooctahedra	MOR	3.34 A mg_PGM_^–1^	86.7% retention after 1000 cycles	^142^
PtPdCdZnCo uf-HEA	Nanoparticle	MOR	2.86 A mg_Pt_^–1^	Strong stability after 7200 s	^143^
Pt_1_–NiCoMgBiSn	Nanoparticle	MOR	35.3 A mg^–1^ (at 2.3 atom % Pt)	Strong stability after 180000 s	^144^
PtRuNiCoFeGaPbW UNWs	Nanowire	MOR	2.61 mA μg_Pt_^–1^	80% retention after 1000 CV cycles	^145^
AuAgPtPdCu	Nanoparticle	CO_2_RR	49.4% CH_4_ FE and 19.9% C_2_H_4_ FE at –0.7 V vs Ag/AgCl	Strong stability after 5 h	^146^
AuAgCuPdPt HEAs	Nanoparticle	CO_2_RR	96.5% CO FE at –0.3 V vs RHE	Strong stability after 50,000 s	^147^
PdCuAuAgBiIn HEAAs	Porous	CO_2_RR	98.1% HCOOH FE at –1.1 V vs RHE	Strong stability after 10 h	^148^
CuBiInZnPd HEAAs	Porous	CO_2_RR	94.7% HCOOH FE at –1.0 V vs RHE	Strong stability after 20 h	^149^
RuFeCoNiCu	Nanoparticle	NRR	38.5% NH_3_ FE at 0.05 V vs RHE	Strong stability after 100 h	^150^
RuFeCoNiMnMo HEA	Nanoflower	NO_3_RR	99.3% NH_3_ FE at –0.6 V vs RHE	Strong stability after 20 cycles	^86^
PdCuNiCoZn	Graphene-like	NO_3_RR	99.0% NH_3_ FE at –0.5 V vs RHE	Strong stability after 50 h	^151^
Fe-CoCuZnCdIn (Fe-HESA NCs)	Nanocage	NO_3_RR	93.4% NH_3_ FE at –0.6 V vs RHE	Strong stability after 150 h	^152^
FeCoNiVAgPd (FL-Ag/HEA)	Nanoparticle	NO_3_RR	92.7% NH_3_ FE at –0.57 V vs RHE	Strong stability after 200 h	^153^
MnFeCoNiCu HEA	Nanofilm	NO_3_RR	94.5 ± 4.3% NH_3_ FE at –0.6 V vs RHE	Strong stability after 250 h	^154^
FeCoNiAlTi (n-HEA)	Polycrystalline	NO_3_RR	95.23% NH_3_ FE at –0.5 V vs RHE	Strong stability after 20 cycles	^155^
PdAuCuIrCo HEA	Nanoparticle-like	C–N coupling: urea electrosynthesis	22.57% urea FE at –0.9 V vs RHE	Strong stability after 6 cycles	^156^
HEA-PdCuAgBiInene	Nanosheet-like	C–N coupling: C_6_H_11_NO electrosynthesis	47.6% C_6_H_11_NO FE at –0.9 V vs Ag/AgCl	Strong stability after 12 cycles	^157^

### 4.1 Fuel Cells

The fuel cell is a type of highly efficient and clean energy device that directly converts chemical energy into electrical energy.^158^ It mainly includes proton exchange membrane fuel cell (PEMFC) with ORR (4e^–^ pathway) and HOR (2e^–^ pathway) as the cathode and anode reactions, respectively, as well as direct-methanol fuel cell (DMFC) represented by MOR (6e^–^ pathway).^159,160^ Among them, ORR and MOR are typical multielectron transfer reactions.

#### 4.1.1 ORR

ORR is the cathode reaction in PEMFCs.^161^ However, the slow kinetics of ORR are the major factor limiting the overall efficiency of PEMFCs.^162^ The ideal reaction pathway for ORR is the complete 4e^–^ transfer, in which O_2_ adsorbed on the catalyst surface ultimately converts to H_2_O. However, the 2e^–^ transfer pathway for H_2_O_2_ formation is a strongly competing pathway, leading to a significant decrease in Faradaic efficiency (FE).^163^ In comparison, the 4e^–^ transfer pathway is more energy efficient and does not produce corrosive products. Pt is a superior active metal for ORR due to its excellent activity and stability.^164,165^ However, the high cost of Pt-based catalysts is a major reason limiting the commercial application of PEMFCs. During the large-scale production, Pt-based catalysts account for approximately 41% of the total cost of fuel cell stacks.^166^ Therefore, by introducing multiple elements into Pt-based catalysts to form stable HEAs, it is possible to significantly reduce the Pt content in the catalyst while improving catalytic activity.^130,141,167,168^ Wang et al. reported that the Pt atomic ratio in as-synthesized PtFeCoNiMn HEAs is only 20%, but the specific activity is 6.5 times that of the commercial Pt/C.^91^ The performance improvement could be attributed to the synergistic effect of multiple sites in HEAs. According to the Sabatier principle, the interaction between the catalyst surface and the reactants should be moderate. Different metal atoms on the surface of HEAs may have different activity properties. Some active sites have strong adsorption and activation for oxygen species, while other sites are more conducive to the dissociation and desorption of intermediates (*OOH, *O, and *OH).^169,170^ Therefore, the high-entropy system can effectively change the binding strength of adsorbed oxygen species and adjust the d-band center.^171^ Liu et al. proposed that the difference in electronegativity of elements in the high-entropy metallenes (HEMs, ultrathin HEA nanosheets) can significantly modulate the local interfacial electric field and O adsorption energy.^172^ Low electronegativity elements Fe/Co/Ni can decrease the d-band center of Pd. Then, the introduction of Pt will further reduce it, thereby weakening the strong adsorption effect on oxygen-containing intermediates. DFT calculations show that the OH* hydrogenation reaction on the PtPdFeCoNiMo HEM (111) surface has a low Gibbs free energy change (0.80 eV). The ORR mass activity as high as 1.40 A mg_Pt_^–1^ was achieved by PtPdFeCoNiMo HEMs at 0.9 V (vs RHE) in 0.1 M KOH electrolyte, which is 21 times that of the commercial Pt/C. Furthermore, Sasaki et al. introduced nitrogen doping into the high-entropy system, and successfully synthesized the N–Pt/HEA catalyst with a Pt-rich shell, a PtCoFeNiCu core, and multiple M–N bonds (Figure 6a).^137^ The catalyst N–Pt/HEA/C exhibits excellent durability, with only a 20.9% loss in mass activity after 30,000 cycles (Figure 6b). For ORR activity, the mass activity is 1.34 A mg_Pt_^–1^ and the specific activity is 1.93 mA cm^–2^ at 0.9 V, which are 7.4 and 6.2 times that of the commercial Pt/C, respectively. DFT calculations showed that the high-entropy effect can significantly weaken the adsorption of *OH on Pt surface. Moreover, the free energy of *O differs significantly between N–Pt/HEA and Pt/HEA surfaces, while the free energy of *OH is similar (Figure 6c). This indicates that nitrogen dopants primarily affect the step of O_2_ reduction to *O.

**Figure 6:**
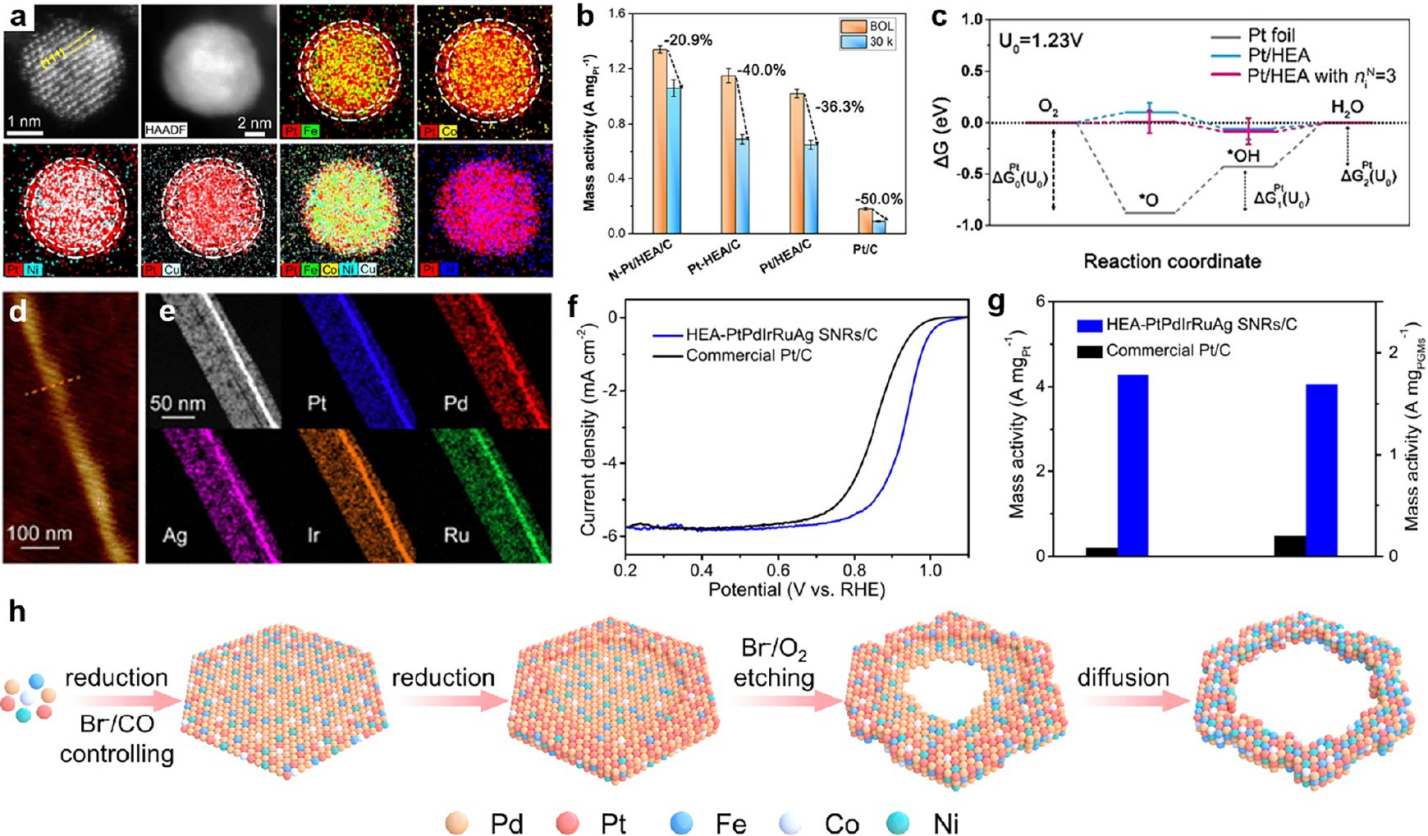
Advanced HEA nanomaterials for electrocatalytic ORR. (a) HAADF-STEM images and EDS elemental mappings of the N–Pt/HEA/C NPs. (b) Variations of mass activity for N–Pt/HEA/C, N-HEA/C, Pt/HEA/C, and Pt/C after 30000 potential cycles. (c) Free energy pathway for ORR at the equilibrium potential (*U*_0_ = 1.23 V) for Pt foil, Pt/HEA, and Pt/HEA. Reproduced with permission.^137^ Copyright 2024, American Chemical Society. (d) AFM image, and (e) HAADF-STEM image and EDS elemental mappings of individual HEA-PtPdIrRuAg SNRs. (f) ORR polarization curves in O_2_-saturated 0.1 M KOH aqueous solution. (g) The column diagram of mass activity for different catalysts at 0.9 V (vs RHE). Reproduced with permission.^138^ Copyright 2022, American Chemical Society. (h) Schematic illustration of the synthesis route of PtPdFeCoNi HEA NRs. Reproduced with permission.^66^ Copyright 2025, Springer Nature.

In addition, the morphology/structure design for ORR high-performance HEA catalysts is very significant. It is worth noting that in ORR, the morphology effect is not only reflected in the increase of specific surface area, but more importantly, it can regulate the exposure of surface crystal faces, the proportion of low-coordination sites, defect types, and local strain states. For special morphologies such as ultrathin 2D structures, subnanobelts, and nanorings, their abundant boundaries and unsaturated coordination sites are more likely to break the adsorption mode of conventional flat surfaces, causing the adsorption energy of oxygen intermediates to deviate from the traditional scaling relationship. In particular, edge defects and compressive strain often help to weaken the excessive adsorption of species such as *OH, ultimately improving ORR kinetics. For instance, Guo et al. developed a series of superthin 2D HEA subnanometer ribbons (SNRs, layer thickness of 0.8 nm) composed of up to eight elements for ORR (Figure 6d,e).^138^ The quinary HEA-PtPdIrRuAg SNRs exhibit a high mass activity of 4.28 A mg_Pt_^–1^ at 0.90 V (vs RHE) in the alkaline electrolyte, which is 21 times that of Pt/C (Figure 6f,g). Site-to-site electron transfer effect between multiple metals with different oxidation/reduction capabilities enhances electrocatalytic performance. Then, Guo’s team further prepared a class of HEA nanorings (NRs) with abundant terrace-type atomic defects to improve ORR performance (Figure 6h).^66^ Most importantly, the asymmetric terrace-type defects can induce attractive interactions and charge redistribution between surface atoms, thereby optimizing the electronic structure of Pt atoms. Therefore, Pt sites on the surface of PtPdFeCoNi HEA NRs with a specific compressive strain could exhibit a downshifted d-band center, and interact less strongly with oxygen-containing adsorbates (i.e., _ad_OH). Specifically, PtPdFeCoNi HEA NRs demonstrate a high mass activity of 0.99 A mg_PGM_^–1^ at 0.95 V (vs RHE). In summary, HEAs have emerged as highly promising ORR catalysts because their diverse local atomic environments enable multisite synergistic catalysis and flexible electronic-structure regulation. Combined with rational morphology and defect engineering, these features allow HEAs to optimize the adsorption/desorption of oxygen-containing intermediates, suppress unfavorable pathways, and simultaneously improve catalytic activity, selectivity, and long-term durability.

#### 4.1.2 MOR

MOR is the core reaction in DMFCs, involving six-electron transfer.^173,174^ While ORR occurs at the cathode, MOR occurs at the anode. MOR can be achieved in both acidic and alkaline electrolytes, but the reaction pathways and products differ. During the MOR process, *CO will be generated as a strongly adsorbed intermediate, occupying active sites. Therefore, CO poisoning of catalysts is a major challenge limiting the large-scale application of DMFCs.^175^ HEAs, with synergistic effects of multiple components, high-entropy effect, and excellent structural stability, have shown great potential for MOR. HEAs can significantly promote the complete oxidation of methanol, avoid CO poisoning, and reduce overpotential.^144,176–178^

However, the components of HEAs should not be chosen arbitrarily, but rather through a systematic design approach. Nam et al. proposed a design route from theoretical modeling to ML prediction, and then to experimental verification.^176^ Based on four PGMs (i.e., Pt, Pd, Rh, and Ru), they systematically screened a series of the fifth element (such as Ir, Os, Au, Cu, Ni, Mo, and Cd) with similar properties to form quinary PtPdRhRuX HEAs. Theoretical calculations revealed that Mo is the relatively optimal active site for MOR. The Mo-containing system exhibited the weakest adsorption tendency for CO, with an adsorption free energy of −1.6 eV, indicating excellent resistance to CO poisoning. The introduction of Mo with high electronegativity led to a charge redistribution between atoms, thus demonstrating promising MOR performance. Furthermore, based on ML prediction, PtPdRhRuMo NPs were successfully prepared on demand. PtPdRhRuMo exhibited a higher MOR activity (12.02 mA/cm^2^) and a lower onset potential (∼0.446 V). In addition, Guo et al. also emphasized the role of Mo and introduced single-atom Mo into high-entropy systems (Figure 7a,b).^114^ Single-atom Mo can effectively modulate the d-band center of adjacent sites, achieving a harmonious balance in reactant dissociation and intermediate adsorption (Figure 7c). As-synthesized Mo-tailored PdPtNiCuZn HEA nanosheets can prevent CO formation and guide the conversion of MOR into a reaction pathway dominated by formate formation. An excellent mass activity of 24.55 A mg_Pt_^–1^ could be realized at the peak potential (Figure 7d).

**Figure 7:**
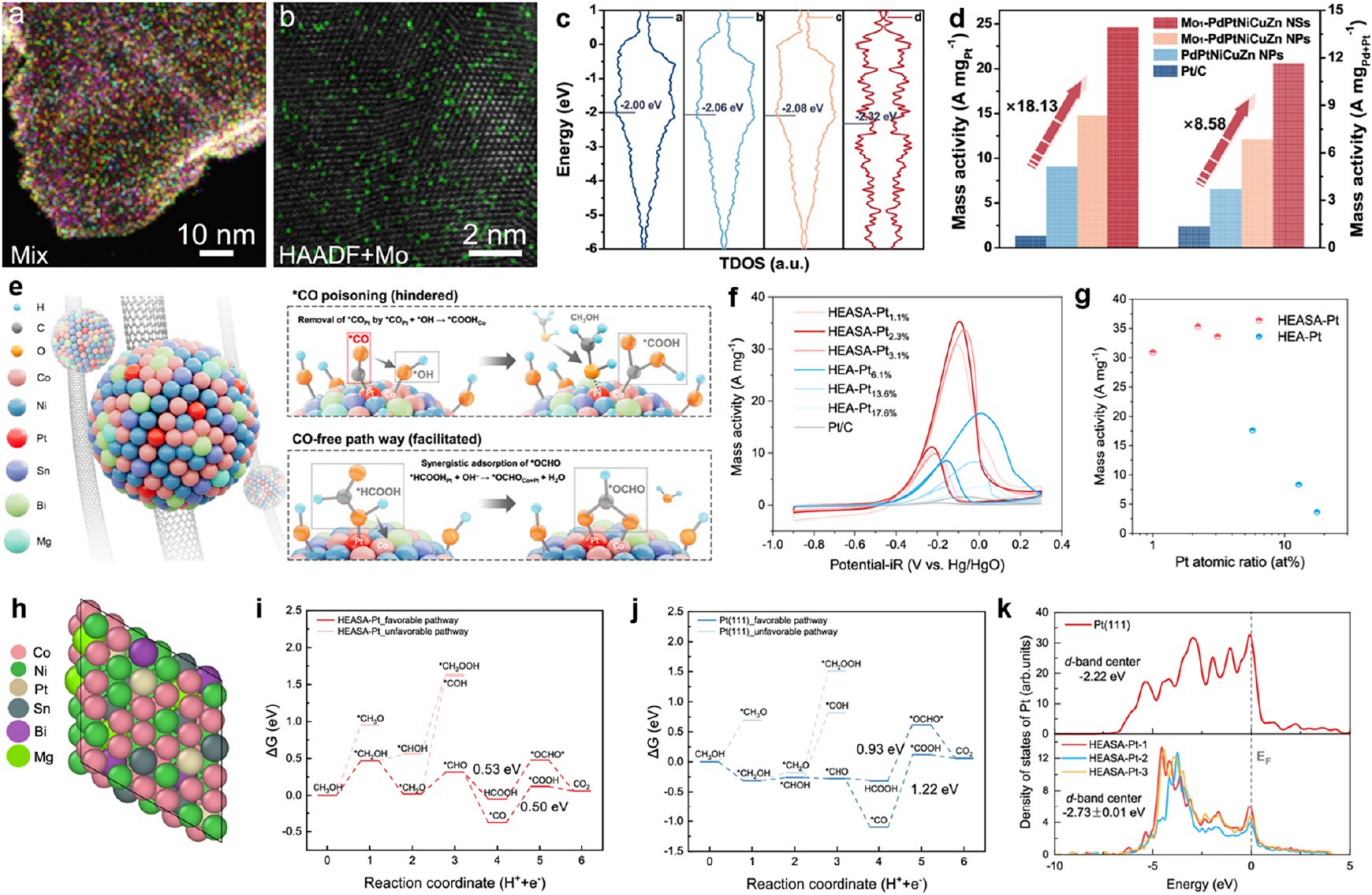
Advanced HEA nanomaterials for electrocatalytic MOR. (a, b) HAADF-STEM images overlapped with the high-resolution EDS elemental mappings of Mo_1_–PdPtNiCuZn SAHEA NSs. (c) The total *d*-density of states in a-strained Mo_1_–PdPtNiCuZn, b-Mo_1_–PdPtNiCuZn, c-PdPtNiCuZn, and d-Pt/C models. (d) A comparison of MOR mass activities. Reproduced with permission.^114^ Copyright 2024, Springer Nature. (e) Schematic illustration of HEASA-Pt NPs on CNTs and the corresponding MOR mechanism. (f) CV curves of a series of HEASA-Pt and HEA-Pt catalysts. (g) The relationship between MOR mass activity and Pt contents of catalysts. (h) A HEASA-Pt surface slab model. (i, j) Free energy pathways of MOR on (i) HEASA-Pt and (j) Pt(111) surface. (k) Partial density of states of Pt for Pt(111) and HEASA-Pt. Reproduced with permission.^144^ Copyright 2025, Springer Nature.

Pt is the dominant active site for MOR. Considering the size effect, HEAs with smaller sizes are more beneficial for improving electrocatalytic activity. For instance, Li et al. reported that PtNiCuMoCoIr nano-octahedra with an edge length of only 2.8 nm demonstrated a good mass activity of 3.34 A mg_PGM_^–1^ in acidic environments.^142^ Ultrafine PtPdCdZnCo HEA NPs (∼2.6 nm) display a high mass activity of 2.86 and 11.1 A mg_Pt_^–1^ in acidic and alkaline conditions, respectively.^143^ However, there is a consensus on the severe CO poisoning phenomenon at Pt sites. Therefore, reducing the Pt content or isolating Pt atoms can effectively suppress the *CO formation pathway. Liu et al. reported the high-entropy alloyed single-atom Pt (HEASA-Pt) (Figure 7e).^144^ It is worth noting that single-atom Pt has no MOR activity. However, dispersing SA-Pt on HEAs shows good MOR performance. Pt_1_–NiCoMgBiSn HEASA-Pt catalysts demonstrate remarkable MOR mass activity of 35.3 A mg^–1^ at the Pt content of only 2.3 at% (Figure 7f,g). DFT calculations revealed that CO adsorption on the HEASA-Pt surface was weakened by more than 0.7 eV compared with that on Pt(111) surface (Figure 7h–j). For the electronic structure, the d-band center of isolated Pt atoms on the HEASA-Pt surface is shifted downward compared with that on the Pt(111) (Figure 7k). Overall, the recent progress in HEA catalysts for MOR highlights that high performance is not achieved by simply increasing compositional complexity, but by rationally engineering the roles of key elements such as Pt and Mo. Pt remains indispensable for methanol activation, whereas Mo is especially valuable for modulating the local electronic structure, weakening CO adsorption, and improving poisoning tolerance. Looking forward, the precise control of Pt content, Pt site isolation, and Mo-assisted pathway regulation will be central to developing next-generation HEA electrocatalysts with high activity, low noble-metal loading, and durable antipoisoning capability.

### 4.2 Electrochemical Synthesis

Today, electrocatalysis technology is no longer limited to energy conversion but has expanded into green electrosynthesis.^179,180^ By driving multielectron transfer reactions with renewable electricity, a series of small molecules, such as CO_2_, N_2_, and NO_3_^–^, can be converted into high-value-added fuels and chemicals.^181–183^ This enables the efficient recycling of carbon and nitrogen resources. Therefore, the application of HEA nanomaterials with unique active sites in electrochemical synthesis has attracted much attention. This section will focus on CO_2_RR, NRR/NO_3_RR, and C–N coupling reactions.

#### 4.2.1 CO_2_RR

Electrochemical CO_2_RR converts the most common CO_2_ into various high-value-added chemicals, such as CO, formic acid, methane, ethylene, and ethanol.^184–187^ CO_2_RR not only helps mitigate the greenhouse effect but also enables the recycling of carbon resources. CO_2_RR is a typical multielectron/proton coupling reaction.^188,189^ It has complex reaction pathways and diverse products, and is also heavily influenced by the electronic structure of catalyst surfaces. The reaction that produces CO and formic acid follows a 2-electron transfer pathway, while the formation of other products, such as CH_4_ and multicarbon (C_2+_) products, follows a multielectron transfer pathway.^190,191^ HER is the main competing reaction, often leading to a decrease in FE.^192^ Many studies report that different metals exhibit different CO_2_RR activities. Cu favors the formation of C_2+_ products.^193,194^ Ag/Au could improve CO selectivity,^195^ while some main-group metals, such as Sn and Bi, favor formic acid production.^196^ Therefore, the HEA nanomaterials with multiple active sites can simultaneously achieve high activity, selectivity, and stability.^148^

However, different local environments within a HEA model can yield distinct intermediate adsorption energies. Therefore, model prediction using ML and high-throughput screening can accurately locate the high-active sites of HEAs.^197^ Sun et al. proposed a strategy combining ML workflows, DFT calculations, and statistical methods to explore the structure–activity-selectivity relationship of HEAs in CO_2_RR, using the (FeCoNiCuMo)_55_ cluster as a model.^198^ Instead of directly evaluating the entire HEA compositional space through brute-force first-principles calculations, they first performed a statistical analysis of the adsorption energies of key CO_2_RR intermediates (including *CO, *CHO, and *H) on a representative HEA cluster model. Based on this, they used a ML-assisted framework to establish descriptors for activity and selectivity. Subsequently, they rapidly screened 26334 HEAs and selected 10 candidate compounds with a balance between activity and selectivity (Figure 8a,b). This workflow is particularly important for HEA catalysis because the enormous configurational complexity of HEAs makes extensive DFT screening difficult, while the combination of ML and DFT provides an efficient route for precisely identifying potential optimal catalysts for CO_2_RR. In another study, Singh et al. used FeCoNiCuMo HEA as the research model and calculated the adsorption energies of three key intermediates, COOH*, CO*, and CHO*, at 1280 surface adsorption sites by DFT.^199^ Based on this, they constructed three independent neural network models to rapidly predict the adsorption performance of different active sites. Figure 8d–f show the comparison between the neural network predictions and DFT calculations of the adsorption energies of COOH*, CO*, and CHO*. As can be seen from the figures, the vast majority of data points fall within the error band of ±0.20 eV. The mean absolute errors of the test set are 0.068, 0.095, and 0.095 eV, respectively, which fully demonstrates that the constructed ML model has high prediction accuracy. In addition, because the interactions of different metal atoms with O atoms vary greatly, they found that COOH* or CHO* can selectively rotate O atoms toward metal atoms that are favorable to the current reaction step, and this “rotation” can break the scaling relationship between the adsorption energies of COOH*, CO*, and CHO* (Figure 8c).

**Figure 8:**
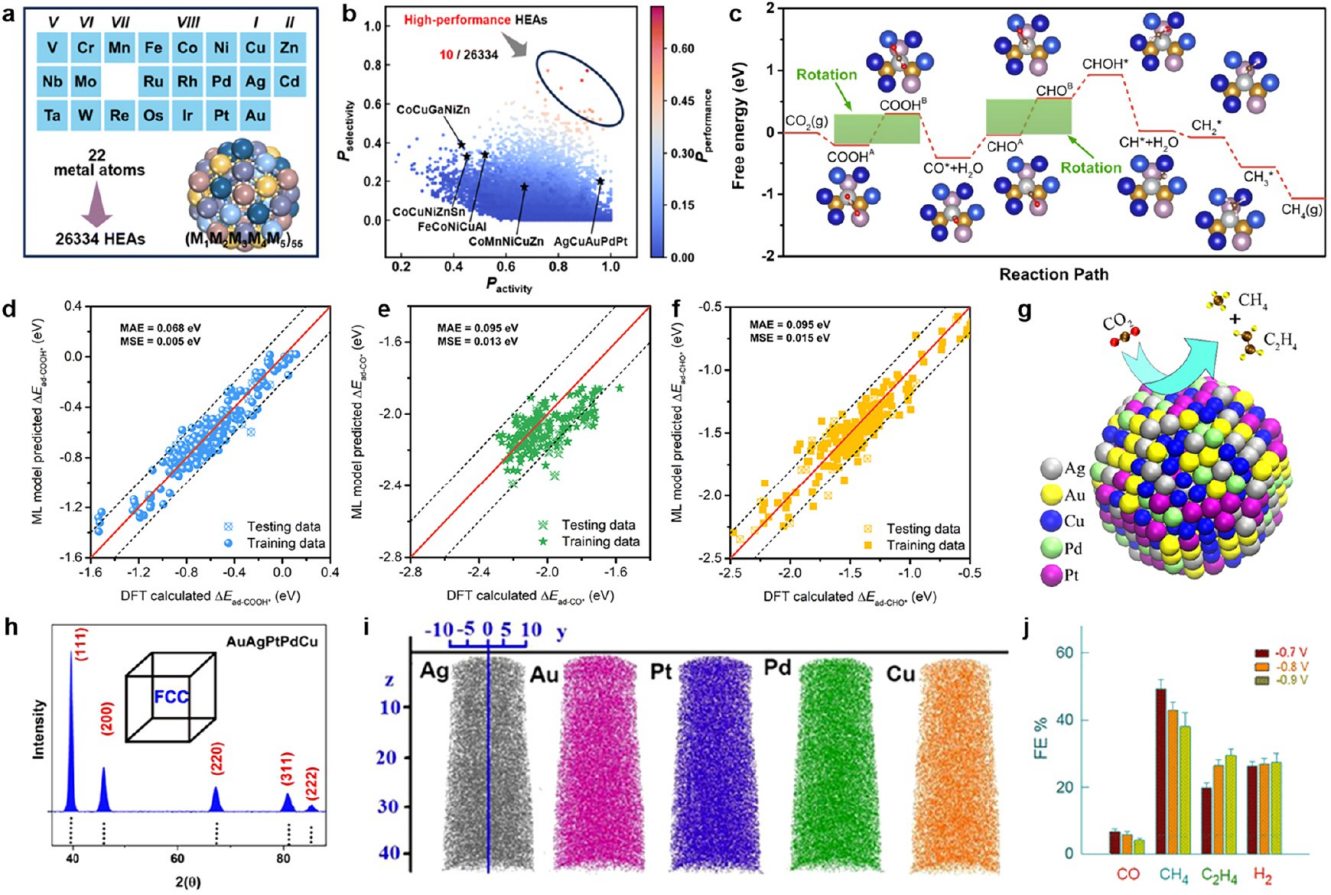
Advanced HEA nanomaterials for electrocatalytic CO_2_RR. (a) All possible five elements combinations of 22 metal atoms. (b) The distribution of *P*_activity_, *P*_selectivity_, and *P*_performance_ for all five element combinations (*P* represents the ratio of active sites with high activity, selectivity, or performance to the total number of active sites). Reproduced with permission.^198^ Copyright 2025, The Royal Society of Chemistry. (c) Reaction process of CO_2_RR on the active site with two rotation regions. Neural network models predict the adsorption energy of (d) COOH*, (e) CO*, and (f) CHO* plotted against DFT calculations. Reproduced with permission.^199^ Copyright 2022, American Chemical Society. (g) Schematic illustration of AuAgPtPdCu HEA NPs and the corresponding CO_2_RR mechanism. (h) XRD pattern of AuAgPtPdCu HEA NPs. (i) The atom probe microscope mapping of Au, Ag, Pt, Pd, and Cu elements. (j) FE of gaseous products during CO_2_RR. Reproduced with permission.^146^ Copyright 2020, American Chemical Society.

The above theoretical calculations help identify the key active sites and electronic structure regulation mechanisms in HEAs. Recently, some experimental works have been reported. Nellaiappan et al. developed the AuAgPtPdCu HEA NPs via melting and cryogrinding for CO_2_RR (Figure 8g).^146^ The XRD pattern of HEA NPs could match well with the *fcc* single-phase structure (Figure 8h). Atom probe tomography revealed that all elements have homogeneous distribution at the atomic level (Figure 8i). The FE of gaseous products is approximately 100% at a relatively low potential of −0.9 V (vs Ag/AgCl). Among them, the formation of CH_4_ and C_2_H_4_ is dominant, with high FE of 49.4 and 19.9% at −0.7 V (vs Ag/AgCl), respectively (Figure 8j).

In summary, HEAs provide a promising platform for CO_2_RR due to their diverse local atomic environments, which offer tunable adsorption energies and multisite synergistic effects. However, to date, research in this field has primarily focused on theoretical calculations, ML-assisted screening, and model predictions, with relatively limited experimental studies. This imbalance may stem from the inherent complexity of CO_2_RR, including its multiple competing reaction pathways, product diversity, and the difficulty in clearly identifying active sites and reaction mechanisms within the complex composition of HEAs. Therefore, future research requires a closer integration of theoretical calculations, high-throughput screening, and in situ/operando experiments to validate predicted catalytic activity, thereby achieving selective and efficient CO_2_ conversion.

#### 4.2.2 NRR/NO_3_RR

The electrochemical N-species reduction reactions (including NRR, NO_3_RR, and nitrite reduction reaction (NO_2_RR), etc.) have attracted widespread attention in recent years.^12,200,201^ These electrochemical methods offer milder reaction conditions, providing a green alternative to the traditional Haber–Bosch reaction. Furthermore, nitrate/nitrite pollutants from industrial wastewater and groundwater across various application scenarios can be effectively removed while producing high-value NH_3_ products.^202,203^ NRR, NO_3_RR, and NO_2_RR are all multielectron/proton coupling reactions with complex reaction pathways. Currently, combining noble metals (such as Ru, Ph, and Pd) and non-noble metals (such as Co, Fe, Ni, and Mo) into binary or ternary alloys can effectively improve catalytic activity.^204–206^ However, the limited active sites and the difficulty in altering the intrinsic properties of metals still constrain the balance between high activity and high selectivity. Therefore, HEAs are expected to be potential catalysts for N-species reduction reactions.

Due to the extreme stability of the N≡N bond and the susceptibility to contamination during experimental detection,^207^ NRR is currently still in the basic research stage.^208,209^ Research on high-entropy nanomaterials for NRR has been rarely reported. Zhang et al. synthesized RuFeCoNiCu NPs via a one-pot wet-chemical method (Figure 9a), and applied HEAs to the NRR.^150^ RuFeCoNiCu/CP demonstrates a NH_3_ yield of 57.1 μg h^–1^ at 0.05 V (vs RHE), and FE of up to 38.5% (Figure 9b). DFT calculations showed that the synergistic tandem effect between multiple elements is key to achieving high performance. Co–Cu and Ni–Ru couples exhibit excellent surface hydrogenation capabilities, with *H tending to adsorb first at these sites (Figure 9c). Then, sufficient *H transfers to t-Fe sites, and activates N_2_ to form NH_3_.

**Figure 9:**
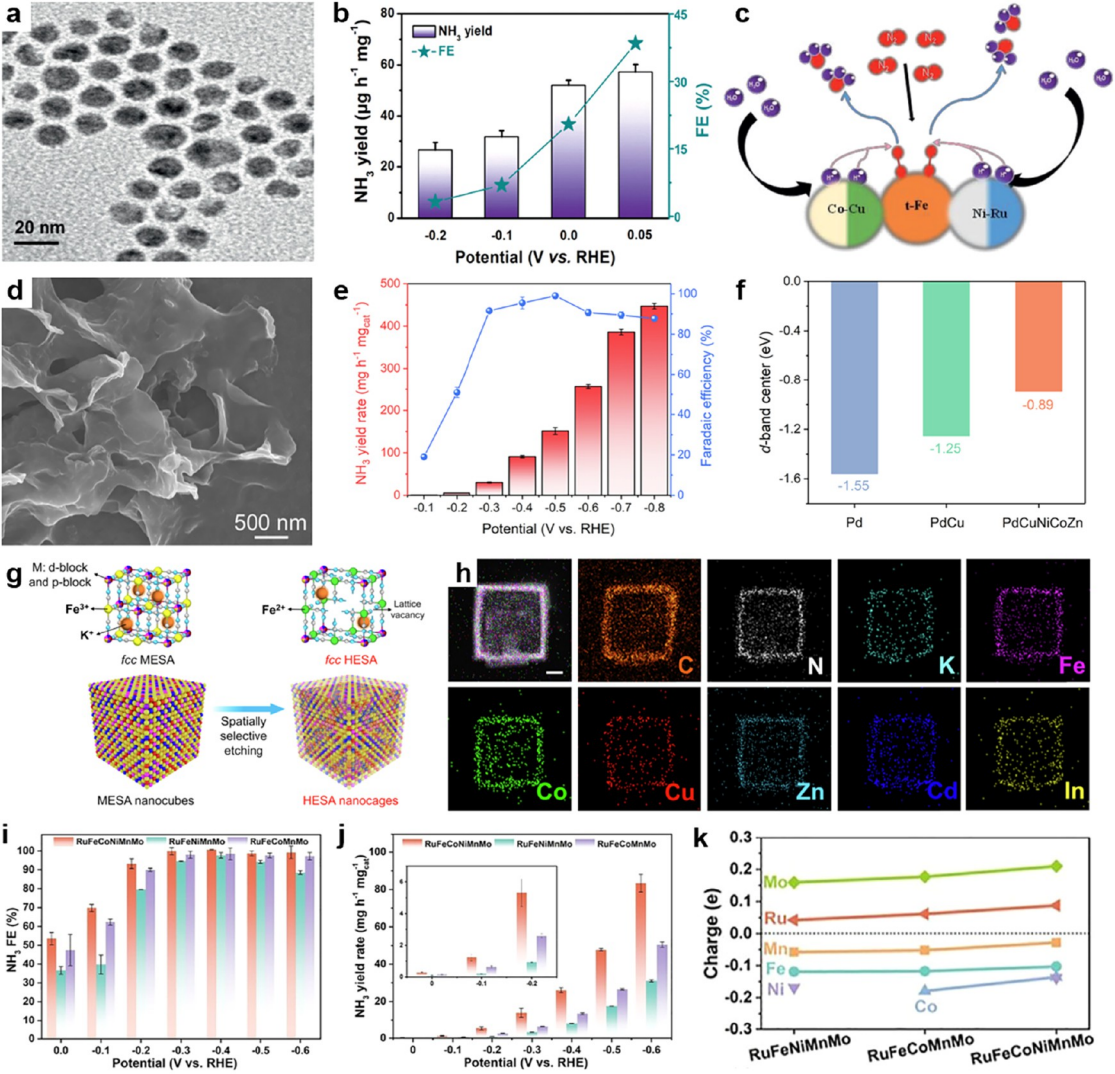
Advanced HEA nanomaterials for electrocatalytic NRR/NO_3_RR. (a) TEM image of RuFeCoNiCu NPs. (b) NH_3_ yield and FE at a series of potentials in 0.1 M KOH. (c) Schematic illustration of a possible reaction pathway to explain the enhanced NRR activity. Reproduced with permission.^150^ Copyright 2021, Wiley-VCH. (d) SEM image of PdCuNiCoZn high-entropy metallenes. (e) NH_3_ FE and mass-normalized yield rate of PdCuNiCoZn. (f) The d-band centers of PdCuNiCoZn, PdCu, and Pd. Reproduced with permission.^151^ Copyright 2025, Springer Nature. (g) Schematic illustration of the synthesis route of HESA NCs. (h) The HAADF-STEM image and EDS elemental mappings of an individual HESA NC. The scale bar is 50 nm. Reproduced with permission.^152^ Copyright 2024, Springer Nature. (i, j) NH_3_ FE (i) and yield rate (j) of *fcc*-*hcp*-*fcc* RuFeCoNiMnMo, RuFeNiMnMo, and RuFeCoMnMo HEAs. (k) The charge distribution comparison. Reproduced with permission.^86^ Copyright 2026, Wiley-VCH.

Compared to NRR, NO_3_RR is easier to achieve and more widely reported.^210–212^ Lu et al. proposed a kind of 2D PdCuNiCoZn HEA metallenes by a facile wet-chemical approach (Figure 9d).^151^ Atomically thin layers of HEAs could achieve the maximized atom utilization. In strongly alkaline conditions, the as-synthesized PdCuNiCoZn HEA metallenes present a yield rate of 447 mg h^–1^ mg^–1^ and near 100% FE for NH_3_ (Figure 9e). DFT calculations revealed that the d-band center of PdCuNiCoZn is closer to Fermi level, which is beneficial to the efficient hydrogenation of *NO, compared with that of Pd and PdCu (Figure 9f). Besides focusing on component design and electronic structure regulation, the morphology and structure of high-entropy nanomaterials also significantly affect NO_3_RR performance. For example, hollow structures can expose more active sites and facilitate the rapid migration of reactants/intermediates/products. Yu et al. introduced a two-step solution-phase synthesis of hollow high-entropy single-atom nanocages (HESA NCs).^152^ The metal atoms of HESA NCs are isolated and covalently coordinated with N or C (Figure 9g,h). Fe-HESA NCs demonstrated a high NH_3_ yield rate of 81.4 mg h^–1^ mg^–1^ at −0.6 V (vs RHE). In another work, *fcc*-*hcp*-*fcc* RuFeCoNiMnMo HEA nanoflowers reported by Meng et al. present a high NH_3_ FE of near 100% and a promising NH_3_ yield rate of 83.35 mg h^–1^ mg_cat_^–1^ at −0.6 V (vs RHE) (Figure 9i,j). Theoretical calculations have revealed that by strong electron coupling induced by high-entropy effect, heterophase *fcc*-*hcp*-*fcc* RuFeCoNiMnMo HEAs have shown strong electronic modulations with charge redistributions. Among all metals, Ru and Mo sites exhibit the most positive charges, while Ni and Co sites attract the most negative charges (Figure 9k). Positively charged Ru sites serve as the main active sites, facilitating the adsorption of NO_3_^–^ in solution, while Ni and Co sites promote the dissociation of H_2_O, providing sufficient active hydrogens for NO_3_RR.

In summary, compared to NRR, which remains in its early stages due to the inertness of N≡N bonds and the difficulty in quantitative ammonia analysis, NO_3_RR demonstrates faster progress and clearer experimental feasibility. Current research indicates that, in addition to compositional design, the electronic structure, morphology, and even phase engineering of HEAs play a crucial role in regulating activity and selectivity. Therefore, future research should focus on elucidating the function of active sites and extending the rational design principles established in NO_3_RR to NRR and related nitrogen species reduction reactions.

#### 4.2.3 C–N Coupling

Recently, the electrocatalytic C–N coupling reaction has emerged as a sustainable synthesis technique. This kind of reaction could directly couple C- and N-species to generate high-value organonitrogen compounds, such as urea, amino acids, formamide, and oximes.^213,214^ These organonitrogen chemicals have important applications in various fields, including agriculture, biology, energy, and chemical engineering. The core of the C–N coupling reaction lies in the simultaneous activation of both C- and N-species on the catalyst surface, promoting the formation of C–N bonds after adsorption with nearby atoms. Therefore, the challenges of this reaction are the unpredictable reaction pathways and the formation of various complex intermediates.

The formation of urea via coupling the small molecule CO_2_ with NO_3_^–^ is a typical C–N coupling reaction.^215,216^ The difficulty of this reaction lies in the fact that the competition of adsorption sites for gaseous CO_2_ is generally weaker than that for NO_3_^–^ present in the liquid phase. NO_3_^–^, while inhibiting CO_2_ coadsorption, is itself completely reduced to NH_3_. Therefore, achieving a balance between the adsorption of CO_2_ and NO_3_^–^ on the catalyst surface is crucial for improving coupling activity. Chen et al. synthesized highly disordered PdAuCuIrCo *fcc*-HEAs and structurally ordered body-centered cubic (*bcc*) AuCuIrCo medium-entropy intermetallic (*bcc*-MEI) via a galvanic replacement approach (Figure 10a).^156^ DFT calculations revealed that *fcc*-HEAs can modulate the desorption energy of *NO_2_ intermediates, and mitigate the strong adsorption of *NO_2_ on the catalyst surface. The *fcc*-HEAs demonstrate a good urea yield rate of 52.43 mmol h^–1^ g^–1^ and FE of 22.57% at −0.9 V vs RHE (Figure 10b,c). Furthermore, the crystal orbital Hamilton population (COHP) index, which can predict the interaction between the adsorbed *CO_2_ and *NO_2_, shows that *fcc*-HEA has a more negative value than *bcc*-MEI (Figure 10d). This indicates that the HEA is more favorable for C–N coupling.

**Figure 10:**
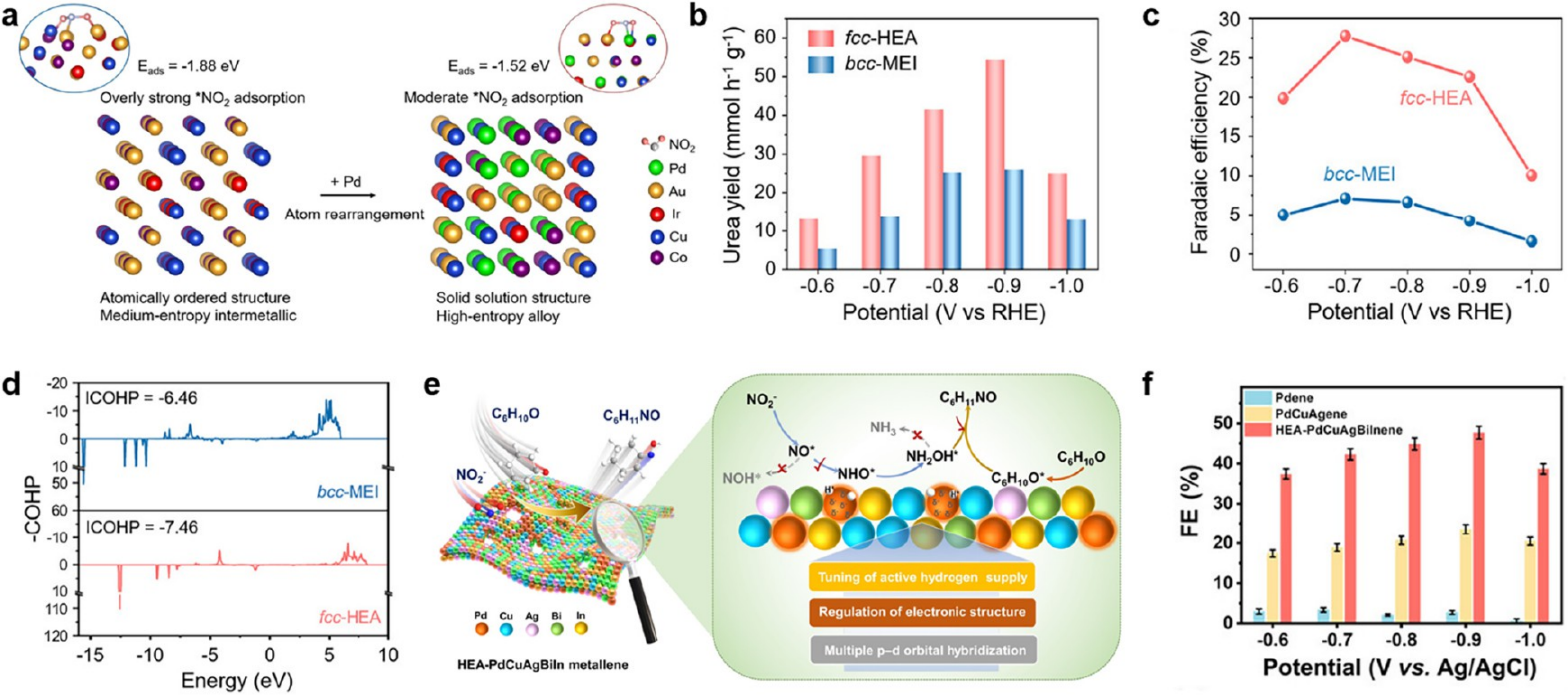
Advanced HEA nanomaterials for electrocatalytic C–N coupling reactions. (a) Structural configurations of MEI and HEA. (b) Urea yield rate and (c) FE of *bcc*-MEI and *fcc*-HEA at different potentials. (d) COHP of *bcc*-MEI and *fcc*-HEA. Reproduced with permission.^156^ Copyright 2025, Wiley-VCH. (e) Schematic illustration of HEA-PdCuAgBiInenes for electrosynthesis of cyclohexanone oxime. (f) Potential-dependent FE comparison over HEA-PdCuAgBiInene, PdCuAgene, and Pdene. Reproduced with permission.^157^ Copyright 2024, Wiley-VCH.

In addition, it is worth noting that hydroxylamine (NH_2_OH) is a key intermediate in the formation of numerous high-value organonitrogen compounds.^217,218^ The unique nucleophilicity of NH_2_OH enables nucleophilic addition–elimination reactions with electrophilic groups (e.g., carbonyl), leading to efficient C–N coupling. Therefore, it is very important to in situ generate NH_2_OH from N-containing molecules and avoid further reduction to NH_3_. At present, by matching appropriate carbon and nitrogen sources, a series of required organonitrogen compounds can be synthesized on demand. NO_3_^–^/NO_2_^–^/NO molecules could react with acids to form the amino acids and amides, and combine with cyclohexanone to form the cyclohexanone oxime. Sheng et al. synthesized porous HEA-PdCuAgBiIn metallene (HEA-PdCuAgBiInene) for the electrosynthesis of cyclohexanone oxime from cyclohexanone and nitrite (Figure 10e).^157^ Orbital hybridization between d-block and p-block metals can modulate the local electronic structure of active sites, thereby regulating the supply of active hydrogen (H*). This causes the nitrite reduction pathway to stop at the NH_2_OH* formation step, inhibiting the subsequent hydrogenation of NH_2_OH*. HEA-PdCuAgBiInene catalysts achieve a high FE of 47.6% and almost 100% yield for the electrosynthesis of cyclohexanone oxime under ambient conditions (Figure 10f).

In summary, the core challenge of electrocatalytic C–N coupling reactions lies in maintaining a kinetically favorable balance between C-species adsorption and N-species activation, while ensuring that key intermediates remain in a coupled active state rather than being completely reduced to undesirable byproducts. In this context, HEAs offer particular advantages because their heterogeneous local coordination environments can modulate adsorption competition, intermediate stability, and hydrogen supply, thereby preventing excessive reduction of key nitrogen-containing precursors and improving coupling selectivity.

## 5 Summary and Outlook

HEAs, as an emerging multielement material system, have demonstrated immense potential in materials engineering and electrocatalysis since their inception. The core characteristic of HEAs lies in their homogeneous solid solutions, which are dominated by high Δ*S*_mix_ and composed of five or more metallic elements mixed in (near-)­equiatomic or nonequiatomic ratios. The HEA system not only overcomes the limitations of traditional single-principal-element alloys but also generates four core effects: high-entropy effect, sluggish diffusion effect, lattice distortion effect, and cocktail effect. These effects collectively endow HEAs with excellent mechanical strength, thermal stability, and tunable electronic structure in electrocatalysis. Especially, in multielectron transfer reactions, HEAs can break the scaling relations of traditional mono/bimetallic catalysts through multisite synergy, simultaneously enhancing activity and selectivity.

The development of HEAs has progressed from bulk to nanoscale, from empirical development to rational design, from zero to multidimensions, and from nonequilibrium synthesis to controllable wet-chemical synthesis. Early bulk HEAs were mainly used for structural materials and functional coatings. The trend toward nanoscale (especially ultrathin 2D structures such as HEMs) has significantly increased specific surface area, exposed more unsaturated coordination sites, and introduced size effects and defect engineering, further amplifying the catalytic potential of the high-entropy effect. The development of wet-chemical synthesis has enabled the high-yield and controllable preparation of anisotropic nanostructures, laying the foundation for the large-scale application of HEAs in electrocatalysis. Benefiting from the synergistic effect between multiple sites to optimize the adsorption/desorption behavior for key intermediates, HEAs have evolved from traditional structural materials into revolutionary materials in the field of electrocatalysis (such as fuel cells and electrochemical synthesis). However, some research challenges and opportunities are still lying ahead, as listed below.

i. *Controllable synthesis of the unique morphology with anisotropy*. Currently, HEAs are mostly NPs, lacking anisotropic structures. This limits their potential in mass transfer and directional adsorption of intermediates for electrocatalysis. Although the controllable synthesis of some specific morphologies via wet-chemical methods has been reported, differences in reduction potentials among different metals make fine-tuning more difficult. Future research should focus on developing precise and controllable anisotropic synthesis strategies. For instance, using surface modifiers and capping agents, template epitaxy, or external field-assisted (electric or magnetic field) induction can achieve multilevel structures in HEAs. Furthermore, the crystal phase (*fcc*, *hcp*, *bcc*, etc.) and the exposure of specific crystal facets significantly affect catalytic activity. Therefore, the rational selection and optimization of crystal facet-selective growth agents can directionally control the crystal facets and phases of HEAs. The anisotropic morphologies can significantly improve mass/electron transport efficiency, expose active sites of specific crystal facets, and adjust the gas–liquid–solid three-phase interface in a flowing electrolyzer.
ii. *Developing mild and facile synthesis methods*. This point is closely related to the previous one. Currently, HEA synthesis largely relies on harsh nonequilibrium synthesis methods, resulting in low yields, high energy consumption, and difficulty in scaling up. While near-equilibrium wet-chemical methods can achieve the synthesis under low-temperature conditions, they may lead to phase separation. Therefore, the development of milder and novel HEA synthesis routes remains urgently needed. Furthermore, introducing multiple non-noble metal elements into noble metal-based materials can significantly reduce material costs and improve sustainability.
iii. *Controllability and reproducibility*. Because the simultaneous introduction of multiple elements with different reduction potentials, diffusion behaviors, and thermodynamic preferences often leads to phase separation, it does not always guarantee the formation of a truly homogeneous high-entropy solid solution. We also emphasize that reproducibility remains a significant issue, especially for nanoscale HEAs prepared via wet-chemical methods. Even minor variations in precursor chemistry, heating/cooling rates, or reaction environments can result in significant differences in elemental distribution and catalytic performance. Furthermore, under electrochemical operating conditions, phenomena such as surface reconstruction, leaching, or localized component redistribution may occur.
iv. *Combination with high-throughput computing*. The compositional possibilities for HEAs are extremely vast, making traditional trial-and-error methods inefficient here. Future research should deeply integrate high-throughput computing (such as DFT, ML, molecular dynamics, and Monte Carlo simulations) with experimental verification to establish a closed-loop process of “computation-screening-synthesis-testing”. ML can be used to predict active-site distribution, adsorption-energy scaling relationships, and stability descriptors, enabling rapid screening superior components. This will significantly shorten the development cycle of novel HEA nanomaterials, ultimately enabling the on-demand synthesis.
v. *Delving deeply into the growth mechanism and reaction pathway*. Due to the complex multicomponent composition, our understanding of the growth process (e.g., elemental diffusion, phase formation) and electrocatalytic reaction pathways (e.g., intermediate evolution, dynamic site reconstruction) of HEAs remains insufficient. In the future, advanced in situ characterization techniques (such as in situ X-ray absorption spectroscopy, in situ TEM, in situ Raman, in situ differential electrochemical mass spectrometry, in situ attenuated total reflection Fourier transform infrared spectroscopy) should be adopted to monitor the alloy growth in real time, as well as the dynamic site/intermediate evolution during reactions (e.g., surface reconstruction after OH adsorption in ORR, CO poisoning pathway in MOR). Special attention should be paid to how the sluggish diffusion effect unique to high-entropy systems ensures the stability of the catalyst, and how multiple sites synergistically break scaling relationships. These mechanistic studies will provide essential guidance for the rational design of advanced HEA nanomaterials. Meanwhile, strengthening the synergistic integration of in situ/operando characterizations and theoretical calculations is encouraged.
vi. *Breaking through the thermodynamic limitations and achieving the coexistence of steady state and metastable states*. Traditional HEAs are thermodynamically constrained, making it difficult to stabilize metastable phases. However, metastable phases may possess higher catalytic activity than the steady state. Future research should overcome thermodynamic limitations to capture metastable structures, and achieve the coexistence of steady and metastable states. This coexistence can produce a synergistic effect: the steady state provides the stability, while the metastable state contributes ultrahigh activity sites. Harsh nonequilibrium methods, due to their rapid heating/cooling properties, hold promise for achieving this goal. Furthermore, introducing multiple elements into a metastable single-metal template to create metastable HEAs is also a feasible strategy. Moreover, the sluggish diffusion effect may be beneficial for stabilizing metastable phases. This research direction is highly challenging and is expected to start a new era for high-entropy materials.

## Vocabulary

High-entropy alloy: a multiprincipal element alloy composed of five or more main elements in an approximately equiatomic ratio or near equiatomic ratio, and forming a simple solid solution structure rather than a complex intermetallic compound
Nonequilibrium state: a physical state in which a system deviates from the thermodynamic equilibrium state during the synthesis of high-entropy alloys
Electrocatalysis: the electrochemical process in which electrode materials or catalysts in the solution can significantly accelerate the charge transfer reaction at the interface between the electrode and the solution, thereby reducing the reaction overpotential and enhancing the reaction efficiency
Faradaic efficiency: the percentage of the total charge passing through the electrode that is used to generate the target product in an electrochemical reaction
Mass activity: the magnitude of current generated by one unit of catalyst at a given potential
